# RNDC-Lab: a real-time calcium imaging-based adaptive neural decoding framework

**DOI:** 10.3389/fnins.2026.1902832

**Published:** 2026-10-09

**Authors:** Kavinda Kehelella, Xiaomin Wu, Hamza Ali, Chen-Wei Yang, Jing Xie, Yaesop Lee, Rong Chen, Shuvra S. Bhattacharyya

**Affiliations:** 1 Department of Electrical and Computer Engineering, University of Maryland, College Park, MD, United States; 2 Department of Robotics, Kwangwoon University, Seoul, Republic of Korea; 3 Department of Diagnostic Radiology and Nuclear Medicine, University of Maryland, Baltimore, MD, United States

**Keywords:** calcium imaging, dataflow, deep neural networks, model updating, real-time neural decoding

## Abstract

**Introduction:**

Real-time neural decoding is critical for precise neuromodulation, which is of increasing interest in the treatment of brain disorders. This paper presents RNDC-Lab, a computational framework for designing, optimizing, and experimenting with real-time, calcium imaging-based, end-to-end neural decoding systems.

**Methods:**

RNDC-Lab integrates calcium imaging preprocessing with adaptive neural decoding using compact Deep Neural Networks (DNNs). RNDC-Lab supports dynamic retraining of a neural decoding model concurrently with inference, thereby providing robustness to changes in the statistical properties of input calcium imaging signals. We integrate NDSEP and NeuroGRS — two recently- developed tools for neural decoding and signal extraction, and optimized generation of DNN models, respectively—into RNDC-Lab. This results in the first real-time neural decoding system based on calcium imaging with a dynamic, DNN-based decoding model. This paper also introduces a number of simulated datasets to assist in system evaluation and experimentation using RNDC-Lab, and more broadly, as resources for experimentation with arbitrary neural decoding models and systems. The experimental capabilities of RNDC-Lab are further enhanced by four new datasets—the Brain Phantom, N80, N90 and Reward Learning (for multiclass classification) datasets—that we introduce in this work as companion resources for working with RNDC-Lab.

**Results:**

To demonstrate the capabilities of RNDC-Lab, we conduct extensive experiments to evaluate the functionality and performance of the proposed framework. The experiments incorporate the aforementioned simulated datasets as well as real calcium imaging data collected from mouse models. Our results demonstrate that the RNDC-Lab system, combined with NDSEP and NeuroGRS, achieves efficient and accurate real-time neural decoding from both real and synthetic calcium imaging data. Moreover, the results demonstrate RNDC-Lab's capability for dynamic model retraining and characterize the associated trade-offs involving decoding performance and execution time.

**Discussion:**

While the components within NDSEP, NGSynth, and NeuroGRS are used to concretely demonstrate the capabilities of RNDC-Lab in this paper, the rigorous dataflow- based architecture of RNDC-Lab enables these components to be selectively replaced with alternative modules that provide the same general functionality using different algorithms or implementations (e.g., a new module for motion correction or model retraining). This makes RNDC-Lab a powerful tool for experimenting with novel aspects of neural decoding in the context of an overall real-time framework.

## 1 Introduction

Miniature calcium imaging (Ghosh et al., 2011; Kerr and Nimmerjahn, 2012; Scott et al., 2013) is a valuable technique for investigating brain circuitry. It allows for optical monitoring of neuronal activity in freely moving mammals, achieving single-cell spatial resolution and sub-second temporal precision. It also facilitates chronic recordings, enabling neural activity to be tracked continuously over the course of several weeks.

Although calcium imaging is an important method for studying neuronal activity, relatively few real-time calcium imaging-based neural decoding systems have been introduced. Previous studies have demonstrated abilities in calcium imaging analysis and neural decoding; however, many of these efforts are developed for specific experimental configurations or focus on individual processing and decoding functions. Consequently, the transition from experimental demonstrations to complete end-to-end real-time neural decoding systems remains insufficiently explored. In particular, a reusable testbench is needed to enable systematic integration and evaluation of components throughout the complete processing pipeline. Such a testbench is important for evaluating system-level efficiency because practical real-time systems must satisfy processing deadlines under limited computational resources. Furthermore, neural signal distributions may change over time, creating a need to update the decoding model without interrupting ongoing inference. These needs motivate a reusable and modular software framework that supports end-to-end system integration and performance evaluation across multiple subsystems, while also supporting the development of real-time online model adaptation mechanisms.

To address these needs, this paper introduces the Real-time Neural Decoding for Calcium Imaging Laboratory (RNDC-Lab), a reusable, dataflow-based software framework for the design, implementation, integration, and evaluation of end-to-end real-time calcium imaging-based neural decoding systems. RNDC-Lab integrates calcium imaging preprocessing with adaptive neural decoding using compact Deep Neural Networks (DNNs). RNDC-Lab can also support the development and evaluation of front-end neural decoding components, which are critical to the design of future neuromodulation systems; the present work focuses on these front-end neural decoding capabilities and does not include integration with a physical stimulation or feedback device. Neuromodulation devices are becoming one of the most powerful tools for the treatment of brain disorders, enhancing neurocognitive performance, and demonstrating causality (Bergmann et al., 2016; Knotkova and Rasche, 2014). The design of neuromodulation systems involves complex trade-offs among (1) the algorithms and machine learning models that are used for neural signal analysis, and (2) the efficiency of the software and firmware that must execute these algorithms in real-time and under the constraints of the targeted processing resources. RNDC-Lab provides a flexible framework for optimized co-design and trade-off optimization across algorithms, models and software implementation, thereby facilitating research and prototyping toward future neuromodulation systems. In addition to being useful for neuromodulation system design, RNDC-Lab is also useful for the design of brain-computer interfaces (BCIs) (Saha et al., 2021). Neuromodulation and BCI are both closed-loop processes requiring real-time neural decoding to provide predictive feedback, and therefore benefiting from the novel co-design capabilities that are provided by RNDC-Lab.

A calcium imaging-based neural decoding system typically consists of two key components. The first is a Real-time Neuron Detection and Activity Extraction (RNDAE) system, which preprocesses the calcium images to generate reliable neural signals for downstream neural decoding tasks. The second is a real-time classification model that predicts behavior labels based on the neural signals produced by the RNDAE system. In RNDC-Lab, we use the Neuron Detection and Signal Extraction Platform (NDSEP) (Lee et al., 2020) as the RNDAE system and NeuroGRS (Wu et al., 2021) as the decoding component. In addition, we employ a new method to integrate these two subsystems, allowing concurrent, real-time neural decoding and updating of adaptive models in response to previously-unseen input distributions.

For preprocessing, motion correction is essential to extract reliable neural signals. However, motion correction algorithms are computationally expensive and challenging to run in real-time. More details about calcium imaging preprocessing and motion correction can be found in Mitani and Komiyama (2018); Lee et al. (2020). NDSEP (Lee et al., 2020) is a well-suited RNDAE system for our RNDC-Lab framework because it focuses on real-time calcium imaging preprocessing, including neuron detection, motion correction, and activity extraction. Furthermore, NDSEP is implemented using dataflow modeling, which offers strong platform adaptability and facilitates integration with other software systems (e.g., see Bhattacharyya et al., 2019).

There are two main challenges for deep learning-based neural decoding models. The first is maintaining high model performance while achieving fast inference speed, since larger DNN models usually predict more accurately but require greater computational resources. The second challenge is to ensure that the model remains adaptive to data distribution drift without interrupting real-time inference in closed-loop applications. Such distribution drift can be biologically motivated by representational drift, where neural activity patterns associated with task-relevant variables change over time, potentially reducing the reliability of fixed decoders (Driscoll et al., 2017; Ratzon et al., 2024).

Deep learning models require significant computational resources for training. For large models, even the forward pass, that is, the inference process, can be too slow for real-time applications. Pruning deep learning models into more compact forms is a promising approach to accelerate inference while maintaining predictive performance (Wu et al., 2021). DNN models require retraining when the input data distribution shifts enough to degrade performance. However, retraining takes significantly longer than inference, and pruning a model could take even more time than training. Therefore, maintaining an up-to-date model without interrupting the real-time inference remains a key challenge. We address this issue in RNDC-Lab by parallelizing the model preparation (updating) and inference processes.

NeuroGRS is an optimized compact DNN model generator (Wu et al., 2021). It can generate compact DNN models from original DNN models without losing significant classification performance. Here, GRS stands for Greedy inter-layer order with Random Selection of intra-layer units, which describes the operation of the core algorithm employed in NeuroGRS. NeuroGRS includes a subsystem called NGSynth with synthesis functionality that converts the generated compact DNN model into a dataflow application graph based on the application programming interfaces (APIs) of the lightweight dataflow (LIDE) framework (Lin et al., 2017). NeuroGRS is an optimized back-end deep learning classifier preparation system. It generates compact DNN models that maintain classification performance comparable to the original (larger) models and that can be deployed under stringent constraints on processing resources and real-time speed. Furthermore, NeuroGRS generates compact DNN models in the LIDE dataflow modeling format, thereby facilitating integration with NDSEP, which also applies LIDE APIs.

Most existing neural decoding systems use microelectrode array technology (Obien et al., 2015) to record neural spiking signals. Furthermore, modern machine learning techniques, especially deep learning, are not fully applied in neural decoding applications. Many current systems still employ traditional approaches such as Kalman filters (Luu et al., 2016). Our work is innovative in introducing a new dataflow-based framework, RNDC-Lab, which supports a neural decoding system built with compact deep learning models as the back-end classifier and raw calcium imaging data as the input source. We also demonstrate that the end-to-end processing time of the entire system is close to the 100*ms* per-frame real-time target, including both calcium imaging preprocessing and deep learning model inference, and quantify the associated deadline-miss behavior. The accurate and real-time decoding performance provided by RNDC-Lab is demonstrated on a low-cost, commodity computing platform, thereby establishing RNDC-Lab as a cost-effective foundation for practical deployment in laboratory testbeds as well as in the prototyping of novel devices for brain-computer interfacing and neuromodulation.

The main contribution of this work is the introduction of RNDC-Lab, an end-to-end neural decoding system for calcium imaging. RNDC-Lab supports parallel machine learning model preparation and inference for real-time calcium imaging-based neural decoding. We integrate NDSEP and NeuroGRS into RNDC-Lab to create the first real-time neural decoding system based on calcium imaging with a dynamic, DNN-based decoding model. NDSEP is used to detect neurons from calcium imaging data, perform motion correction on each frame, and extract neural signals for decoding. NeuroGRS is applied to generate compact DNN models for decoding these signals. By carefully leveraging dataflow modeling, RNDC-Lab achieves an efficient system implementation leading to reliable real-time operation and enabling extensibility to incorporate alternative algorithms and implementations for specific aspects of the neural decoding process. RNDC-Lab also enables model updates during runtime through dynamic loading and dataflow-based modules for correct and efficient communication between processors.

## 2 Related work

In this section, we summarize representative neural decoding systems in the existing literature and compare them with our proposed neural decoding framework, RNDC-Lab, as well as with the neural decoding subsystems — NDSEP, NeuroGRS, and NGSynth—that are integrated within RNDC-Lab. The major subsystems integrated into RNDC-Lab have been evaluated independently in prior work. NDSEP was benchmarked against established calcium-image motion-correction and neuron-detection methods, including CaImAn-NoRMCorre, SIFT, CaImAn-CNMF, and CellSort (Lee et al., 2020). On the real ALM dataset, NDSEP achieved a recall of 72.46%, a precision of 69.44%, and an F1 score of 70.92%, while processing the complete motion-correction, preprocessing, neuron-detection, and signal-extraction pipeline in 20.67 ms per frame. NeuroGRS was evaluated on nine real calcium-imaging datasets using original fixed-weight MLP and CNN models, as well as the NWM and RRS pruning baselines (Wu et al., 2021). The resulting compact models retained most of the prediction accuracy of the original models while substantially reducing FLOP and parameter counts; on a Raspberry Pi Zero, pruning reduced the inference time of the two evaluated CNN models by 11.631 ms and 11.108 ms, respectively. These prior evaluations establish the accuracy and computational efficiency of the preprocessing and compact-decoder subsystems integrated into RNDC-Lab.

Wu and Hatsopoulos demonstrated the use of linear regression and Kalman filter models for real-time adaptive neural decoding of hand motions (Wu and Hatsopoulos, 2008). Additionally, they developed a recursive update approach. The novelty of RNDC-Lab compared to that of Wu and Hatsopoulos includes two major aspects: (1) RNDC-Lab collects neuronal signals from calcium imaging frames, and (2) RNDC-Lab incorporates more sophisticated techniques for signal processing and machine learning, including optimized DNN models.

Shin et al. introduce real-time neural decoding for finger movement applications (Shin et al., 2009). The core method for decoding neural signals is based on maximum-likelihood inference. Compared to their work, the novelty of RNDC-Lab lies in applying DNNs for real-time neural decoding. Moreover, we apply network pruning to reduce model size and leverage dataflow modeling with LIDE to implement the system more efficiently.

Two general-purpose neural decoding platforms are introduced in Glaser et al. (2020) and Meyers (2013). These platforms support neural decoding using various machine learning classifiers that can be applied to neuronal spiking signals. Nasiotis et al. (2019) introduced an open-source software platform that provides data analysis, data management, and multi-modality integration solutions for invasive neurophysiology. This work shares common features with RNDC-Lab in that both are end-to-end systems—that is, they collect neural signals and perform subsequent analysis. However, the platforms presented in Glaser et al. (2020) and Meyers (2013) are not designed for real-time neural decoding.

Recent studies have demonstrated several forms of real-time calcium imaging processing, neural decoding, and closed-loop feedback. Taniguchi et al. (2021) developed Carignan, an open-source system that performs online calcium image processing, detects predefined synchronized neuronal firing patterns, and triggers an external device. However, the firing patterns must be manually defined in advance, and the system does not perform behavioral decoding or runtime model updating. Liu et al. (2021) developed an optical brain-computer interface that integrates image acquisition, manually selected neuronal features, SVM training, and online lever-press decoding. However, the system relies on manual neuron selection and a fixed, task-specific SVM model, and it does not support runtime model adaptation. Chen et al. (2023) introduced an end-to-end FPGA-based pipeline that integrates motion correction, image enhancement, trace extraction, neural-network decoding, and a hardware feedback interface, achieving deterministic sub-millisecond decoding latency. However, its primary emphasis is the dedicated hardware implementation and optimization of a predefined pipeline rather than flexible subsystem replacement and runtime model adaptation. Compared with these systems, RNDC-Lab not only addresses their limited flexibility and lack of runtime adaptation, but also provides a reusable and modular dataflow-based software framework that supports platform retargeting, replacement of processing and decoding modules, concurrent model preparation and inference, and dynamic loading of updated decoding models. Additionally, RNDC-Lab employs compact neural networks to reduce inference computation requirements and to support future adaptation to more resource-constrained computing platforms.

## 3 Methods

### 3.1 RNDC-Lab framework

We propose RNDC-Lab as a real-time, end-to-end, calcium imaging-based neural decoding framework. As shown in Figure 1, RNDC-Lab receives calcium imaging frames as sensing data and processes them through motion correction, neuron detection, signal extraction, and signal stabilization. The resulting neural signals are then sent to both the real-time inference path and the parallel model-updating path. In the updating path, the corresponding behavioral labels serve as ground-truth values for accumulating training data and generating a new model, which is subsequently loaded into the inference subsystem. The current model produces the predicted output values, such as decoded kinematics or behavioral choices.

**Figure 1:**
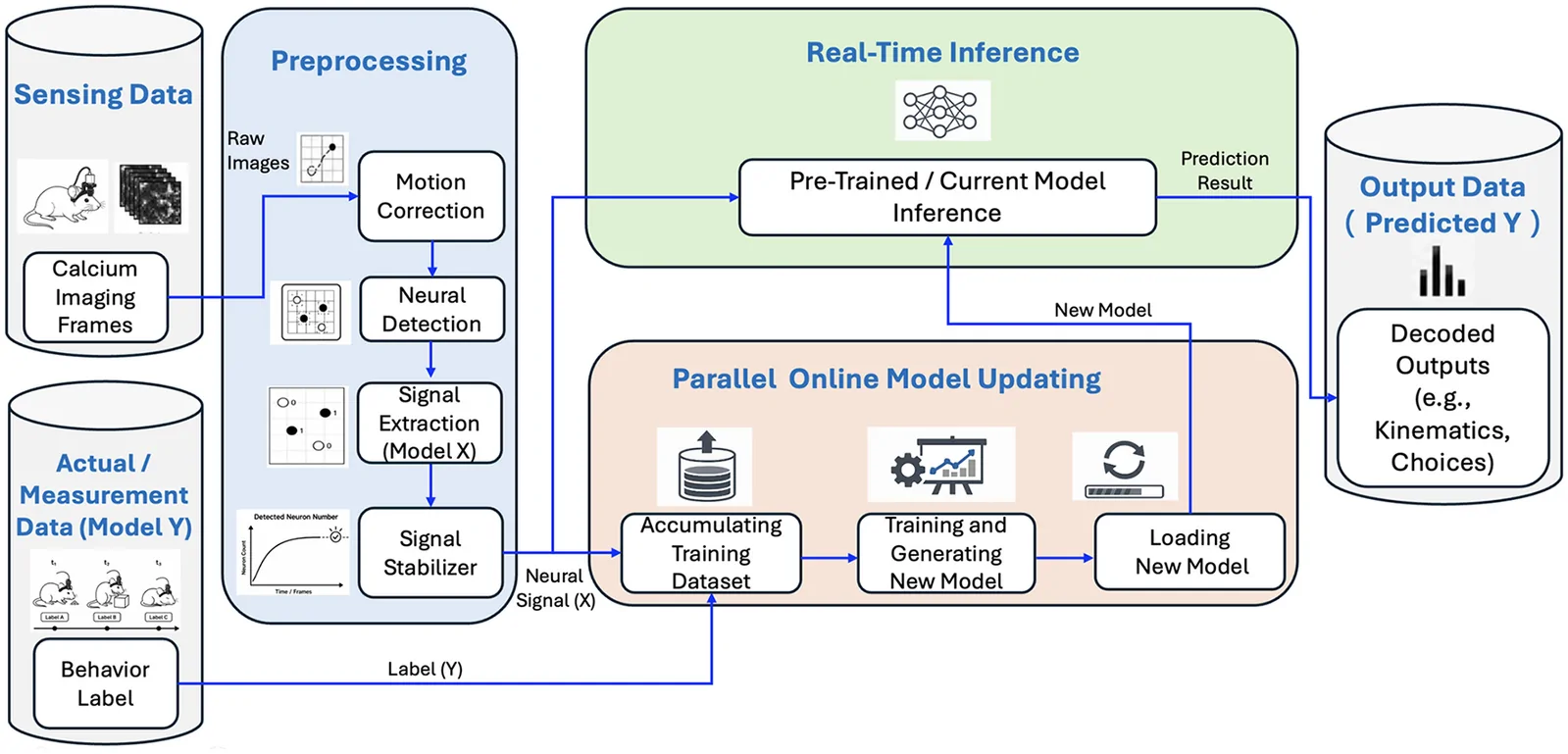
Overview of the RNDC-Lab workflow for real-time calcium imaging-based neural decoding with parallel online model updating.

The system integrates three major subsystems: the preprocessing subsystem (RNDAE), the decoding model preparation subsystem, and the model inference subsystem. RNDC-Lab includes both an initialization stage and an operational stage. The neural decoding system is initialized after a short period following system startup. During initialization, the neural signals extracted by the RNDAE system must stabilize, and the decoding model must be fully prepared and synthesized into the inference subsystem. Combining the initialization and operation stages is important because this design helps avoid pausing real-time data recording for initialization and ensures that parameter initialization remains consistent across different subsystems. This design is also compatible with pre-trained models—that is, models trained offline using prepared data before the input data stream is applied to the system for inference. If a DNN-based neural decoding model is available, the system will automatically skip the initialization stage. Figure 2 illustrates the hierarchy across reusable subsystems and the RNDC-Lab-specific integration and orchestration layers.

**Figure 2:**
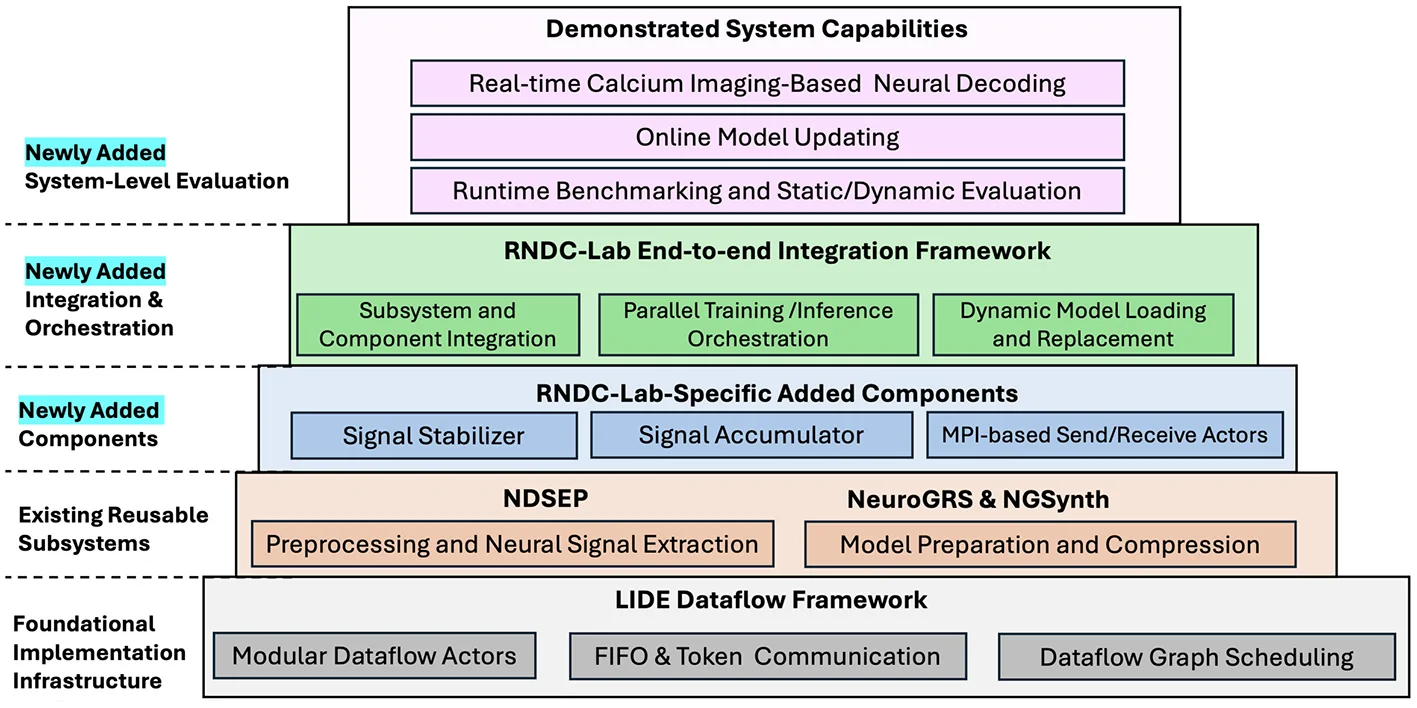
Hierarchy of the RNDC-Lab framework relative to reusable dataflow and neural decoding subsystems. RNDC-Lab builds on LIDE, NDSEP, NeuroGRS, and NGSynth while adding system-level orchestration, signal stabilization, signal accumulation, MPI-based send/receive actors, parallel training/inference coordination, dynamic model loading, and runtime benchmarking capabilities.

The motivation for incorporating a model updating mechanism without interrupting real-time inference is to maintain the performance of the back-end classifier when the input data stream undergoes potential distribution shifts. Machine learning models are trained on the distribution of the training dataset, and their performance can degrade significantly when the distribution of unseen data differs substantially from that of the training data (Bhattacharjee et al., 2020). It is difficult to construct a general training set that covers all potential data generation processes. This typically requires collecting a large amount of high-quality training data. DNN models used for prediction on data streams often suffer when trained on a limited dataset and deployed on a semi-infinite data stream, whereby *semi-infinite*, we mean that the stream begins at some well-defined point in time and there is no bound available a priori on when the input stream will end. Therefore, a model updating mechanism is essential for machine learning applications involving streaming data.

The common approach to addressing this problem in non-real-time applications is to retrain the model periodically and update it offline. However, this strategy requires stopping the currently running inference model during training and subsequent deployment of the new model. It often involves human intervention to manually deploy the newly trained inference model. These steps interrupt real-time operation, especially when frequent updates are needed. Therefore, this approach is unsuitable for real-time applications.

Online learning methods (Sahoo et al., 2017) perform inference on incoming data batches from the stream and then retrain the current model using those batches for the next iteration. Although this approach does not halt software execution, it still consumes part of the inference cycle for training, rather than allowing continuous inference. Moreover, if the batch size becomes too large, the training phase may take too long, making it impossible for the system to meet real-time requirements.

RNDC-Lab addresses this issue by parallelizing training and inference. In particular, one group of processors is utilized to perform continuous inference while another group handles training in the background. Furthermore, RNDC-Lab applies dynamic loading techniques (Beazley et al., 2001) to seamlessly replace the previously trained model with the newly trained one, without interrupting the real-time inference cycle.

Dataflow provides a valuable foundation for the design and implementation of novel signal and information processing systems under complex constraints (Bhattacharyya et al., 2019). When dataflow is used as an abstraction for design of stream processing systems, applications are represented as directed graphs, called *dataflow graphs* (Lee and Parks, 1995). Vertices in dataflow graphs, called *actors*, represent basic functional units that often can be reused in other application graphs, such as neuron detection, motion correction, and signal stabilization. Each edge in a dataflow graph represents a first-in, first-out (FIFO) buffer that stores data as it passes from the output of one actor to the input of another. Each unit of data within such a buffer is referred to as a *token*. Tokens can be of arbitrary complexity in dataflow-based applications—for example, a token can correspond to an integer value, floating point value, a vector, an image or a video clip.

A dataflow actor executes as a sequence of *firings*, where each firing can be viewed as a discrete unit or quantum of the actor's computation. A firing can be executed when an actor has sufficient data, where this notion of sufficiency is defined precisely as part of the design of the actor. The actual time at which different actors execute is determined by a subsystem called a *scheduler*, which is not part of the dataflow graph model. This separation of concerns between functional specification (provided by the dataflow graph) and dispatching of component executions (provided by the scheduler) is an important feature of dataflow-based design processes. For more details on the form of dataflow that we apply in this work, we refer the reader to Lee and Parks (1995); Bhattacharyya et al. (2019).

RNDC-Lab integrates three subsystems—NDSEP, NeuroGRS, and NGSynth. We adapt NDSEP as the front-end RNDAE subsystem, NeuroGRS as the back-end subsystem for machine learning classifier preparation, and NGSynth as the back-end subsystem for inference. Our goal is to achieve a real-time integrated system. The real-time requirement for the neural decoding system that we utilize for our experimental demonstration is 10 Hz, meaning the entire system must process each calcium imaging frame within 100 ms. To reduce inference computation costs, we use a compact neural network. During the initialization stage, we enable NeuroGRS's structure search functionality to identify a compact neural network architecture for use in the operation stage. In the operation stage, NeuroGRS creates new models based on the fixed structure found during initialization.

By integrating the aforementioned three subsystems, RNDC-Lab addresses three major challenges: first, how to coordinate the training and inference stages of NeuroGRS with the NDSEP system during runtime; second, how to perform training and update the decoding model without interrupting real-time inference; and third, how to integrate the newly compiled LIDE dataflow graph of NGSynth inference into the dataflow application graph of the entire system during runtime.

The RNDC-Lab framework, NDSEP and NGSynth are implemented using the LIDE dataflow package (Lin et al., 2017), featuring well-organized module structures and precisely-defined interfaces between dataflow actors. As a result, NDSEP and NGSynth can be systematically integrated into the RNDC-Lab framework as new LIDE dataflow subgraphs. Furthermore, they can be conveniently extended with new functionalities by adding LIDE dataflow actors or by combining dataflow application graphs as subgraphs.

### 3.2 The NGSynth subsystem

NGSynth is a dataflow-based deep neural network inference system designed for real-time inference. It is fully implemented using dataflow modeling and can convert any Multi-Layer Perceptron (MLP) or Convolutional Neural Network (CNN) saved in TensorFlow format into a LIDE dataflow application graph. NGSynth treats neural network layers as basic units and implements fundamental LIDE dataflow actors for dense layers, convolutional layers, various activation layers, flatten layers, softmax layers, and other essential components needed to assemble MLPs and CNNs. The synthesis functionality of NGSynth determines the number of actors, actor types, and parameters within each actor—for example, the number of nodes in a dense layer. It then automatically creates a LIDE dataflow application graph and compiles it into an executable or library. NGSynth is under active development to support additional model types such as RNNs and LSTMs, as well as to accommodate various input sources, including PyTorch-saved models and models specified using the dataflow interchange format (DIF) tool from the University of Maryland (Lin et al., 2017).

NGSynth is well-suited for building dataflow-based deep learning application systems, as it can generate models in the form of LIDE dataflow application graphs. These deep learning models can then be connected to other LIDE dataflow application graphs to construct larger systems. For example, in this work, we connect the LIDE-based dataflow graph generated by NGSynth for a compact MLP model with the LIDE-based dataflow application graph of the overall RNDC-Lab framework. Another advantage of NGSynth is that it generates LIDE-based dataflow application graphs in the C programming language. As a result, DNN models implemented with NGSynth have a runtime advantage on many platforms over those built using Python-based packages. In particular, the NGSynth-based DNN inference system can be deployed on platforms with real-time requirements and limited computational resources.

To demonstrate the use of NGSynth, we take the classifier employed in this work as an example. NGSynth generates a LIDE-based dataflow application graph of a compact MLP model, which is shown in Figure 3. The number of nodes inside each dense layer actor is decided by the final compact model provided by NeuroGRS. The MLP model implemented using NGSynth in Figure 3 involves four types of NGSynth actors: the *Loader* actor, *denseLayer* actor, *Relu* actor, and *Softmax* actor. Two FIFOs *FIFO*_*In* and *FIFO*_*Out* connect directly to the LIDE dataflow graph of the RNDC-Lab framework. The FIFO *FIFO*_*In* buffers input neuron spiking signals from the RNDC-Lab framework, and *FIFO*_*Out* is used to store output decoding results that are sent back to the enclosing RNDC-Lab framework for further processing. The *Loader* actor only executes once when each new model is loaded. The *DenseLayer*, *Relu*, and *Softmax* actors are invoked once per input sample during inference. *DenseLayer*, *Relu*, and *Softmax* support intra-actor parallel computing with OpenMP-based CPU (Dagum and Menon, 1998) computation, as well as GPU computation. In the experiments presented in this work, we utilize the CPU computation mode of NGSynth.

**Figure 3:**
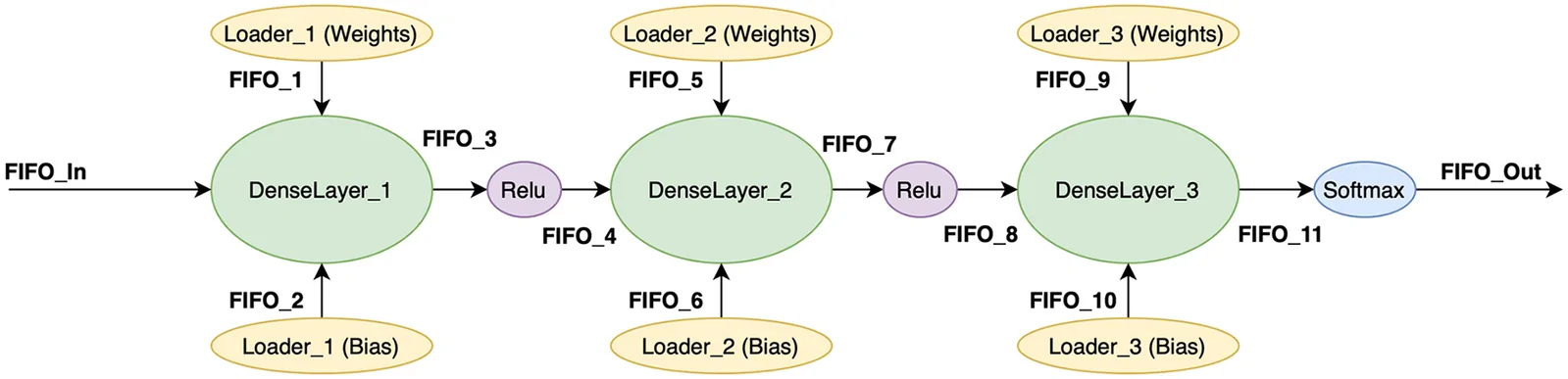
Example of an MLP model implemented using the NGSynth subsystem along with LIDE.

### 3.3 RNDC-Lab framework with dataflow modeling

As shown in Figure 4, the RNDC-Lab framework consists of two LIDE dataflow graphs running in parallel. The framework includes the following major functional units: multi-core communication, neuron signal preparation, decoding model preparation, and decoding model inference. The multi-core communication function is handled by the *snd*_*dsg*_*mpi* and *rec*_*dsg*_*mpi* actors. The neuron signal preparation unit consists of the RNDAE, *signal*_*stabilizer*, and *signal*_*accumulator* actors. The RNDAE actor can wrap any RNDAE subsystem for generating neuron signals from calcium imaging. We wrapped NDSEP inside the RNDAE actor and labeled it as the *RNDAE*_*NDSEP* actor. The decoding model preparation unit comprises the *model*_*preparation* actor and the *synthesis* actor. The *model*_*preparation* actor can wrap any model preparation subsystem. In this work, we integrate NeuroGRS into the *model*_*preparation* actor and denote it as *MP*_*NeuroGRS*. The decoding model inference unit comprises the *model*_*inference* actor, which can wrap any model inference subsystem. Here, we wrap NGSynth within the *model*_*inference* actor and label it as *MI*_*NGSynth*.

**Figure 4:**
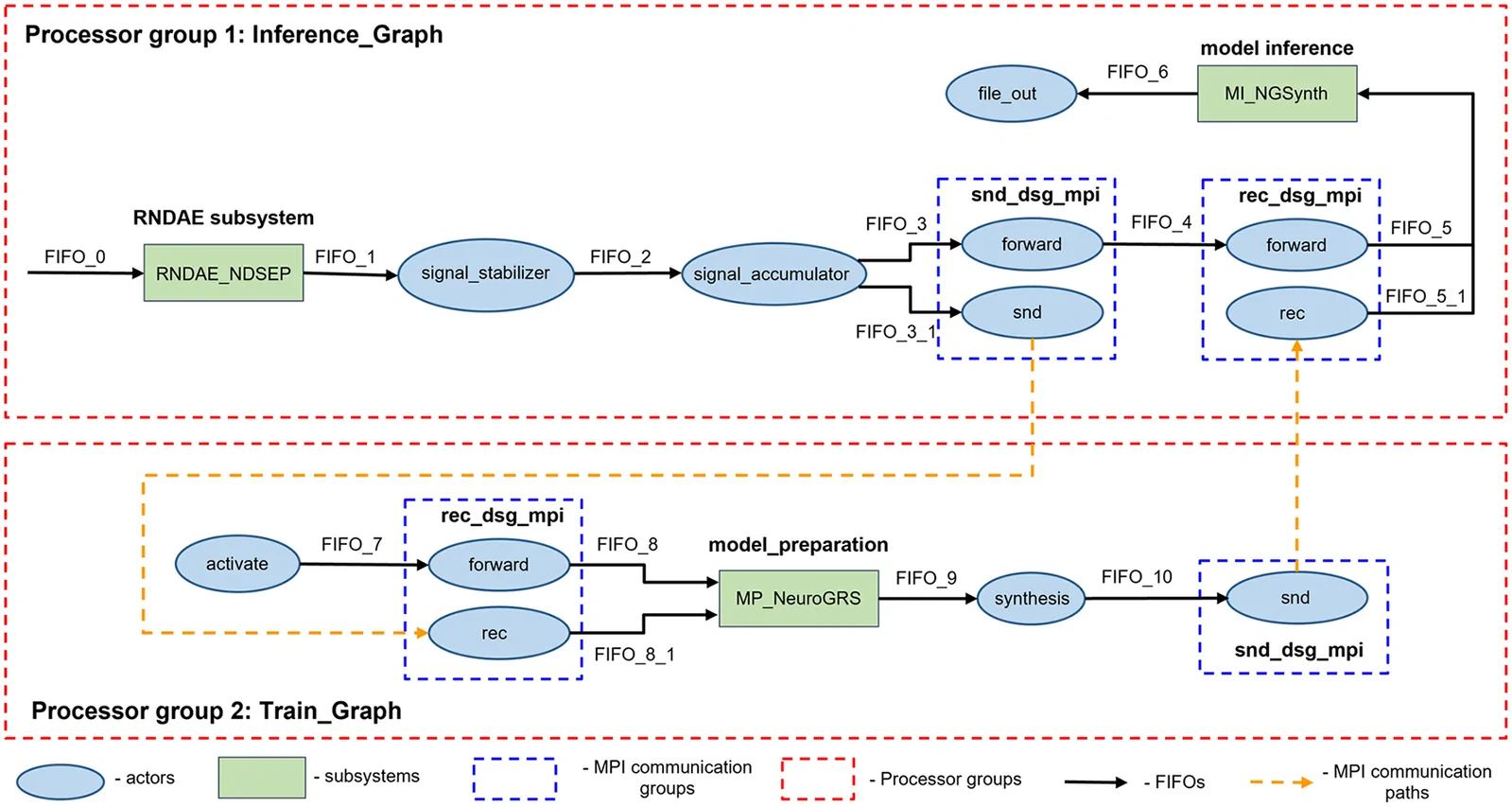
Dataflow modeling of the RNDC-Lab framework with NDSEP, NeuroGRS, and NGSynth as subsystems. Blue ellipses represent actors, while green rectangles represent subsystems containing multiple actors. Blue dashed rectangles denote MPI communication groups, and red rectangles represent processor groups. Black solid arrows indicate FIFO connections, while orange dashed lines represent MPI communication paths (i.e., remote communications between processor groups).

The RNDAE system, such as NDSEP, often produces neuron signals with varying numbers of detected neurons during the early phase of initialization — that is, in frames captured shortly after the system starts. The *signal*_*stabilizer* actor helps stabilize the spiking signals from *RNDAE*_*NDSEP* and ensures that both the training and testing data have a consistent number of features for the back-end decoding model generated by *MP*_*NeuroGRS*. The *signal*_*accumulator* actor collects batches of samples from the incoming data stream, which are then used by *MP*_*NeuroGRS* to train a decoding model.

As shown in Figure 4, *signal*_*stabilizer* has one input FIFO *FIFO*_1 and one output FIFO *FIFO*_2. Each token passed through *FIFO*_1 is a vector of *D* features, where *D* is the number of detected neurons, and each feature represents the neural signal from a distinct neuron. During the early frames, the neuron detection process continues to identify new neurons, which are added incrementally, frame by frame. The main function of the *signal*_*stabilizer* actor is to monitor the input tokens over a window of *F*_*stable*_ frames. It begins forwarding input signals to *FIFO*_2 once the number of detected neurons becomes consistent within that *F*_*stable* frame window. After this stabilization point, any newly detected neurons are discarded. However, this is rare if the *F*_*stable* hyperparameter is set sufficiently large to allow *RNDAE*_*NDSEP* to identify all detectable neurons from calcium imaging data. The shifts in the position of neurons caused by camera vibration during calcium imaging are corrected by the motion correction module within *RNDAE*_*NDSEP*. We assume that the total number of detectable neurons remains consistent across calcium imaging frames within the same experimental setup. The *signal*_*stabilizer* must be recalibrated if the experimental conditions change.

As shown in Figure 4, *signal*_*accumulator* has one input FIFO *FIFO*_2 and two output FIFOs *FIFO*_3_1 and *FIFO*_3. The tokens that are stored in *FIFO*_2 are stabilized neuron signals. The *signal*_*accumulator* actor collects *A* frames as a batch and outputs the batch of samples using the *FIFO*_3_1 FIFO. Here, *A* is the number of samples that is used to re-train the neural decoding model concurrently with inference using the current model version. The *signal*_*accumulator* actor will only forward samples to the training graph (via *FIFO*_3_1) when the collected samples on its input reach the preset amount *A*. While accumulating samples from *FIFO*_2, the *signal*_*accumulator* actor also forwards tokens encapsulating neural signals from a single frame to the FIFO labeled *FIFO*_3. The batch of *A* collected samples is used as the training set for *MP*_*NeuroGRS* to produce a new compact DNN model, while the forwarded samples of individual frames are taken as the input for *MI*_*NGSynth*. If more than *A* samples accumulate while training on an earlier window of *A* samples is in progress, then earlier samples are discarded for subsequent training; the most recent *A* samples are utilized when a new round of training begins.

To enable supervised model-retraining within RNDC-Lab, not only do we need to send recently-acquired samples to the training subsystem, but also we need to provide the corresponding labels. We assume that the true label *y*^*t*^ of a given instance *x*^*t*^ is available before the arrival of *x*^*t*+1^, enabling us to train models based on streaming data. This is a widely-used assumption in stream classification (Oza and Russell, 2001; Gomes and Enembreck, 2014; Bifet et al., 2010), as it is consistent with many practical applications. In applications where this assumption is not satisfied, RNDC-Lab can be utilized by disabling the training subsystem once inference begins, and utilizing the pre-trained neural decoding model as a fixed-parameter model.

As shown in Figure 4, the compact decoding model represented by a LIDE dataflow graph is wrapped inside the *MI*_*NGSynth* actor as the model inference subsystem. The *MI*_*NGSynth* actor has two input FIFOs: *FIFO*_5 and *FIFO*_5_1. *FIFO*_5 transfers tokens as single-frame data samples, i.e., stabilized neuron signals from one frame. *FIFO*_5_1 transfers tokens as binary values, which indicate whether the model preparation by *MP*_*NeuroGRS* and *synthesis* is completed and the newly generated compact decoding predictor (i.e., the updated parameter set) is ready to be loaded. After the *MI*_*NGSynth* actor finishes inference on one sample, a binary value will be returned and output to the *FIFO*_6 FIFO. This binary value corresponds to the predicted behavior label for the sample. In Section 4, most behavior labels are binary-valued; however, RNDC-Lab also supports multiclass classification, as demonstrated in Section 4.5.1. The framework can be configured for multiclass classification through suitable replacement of the training and inference subsystems.

Inter-processor communication is needed to realize the parallel execution of *Inference*_*Graph* and *Train*_*Graph*. Therefore, we adapt concepts of *snd* and *rec* actors from the Dataflow Schedule Graph (DSG) (Wu et al., 2011) model and implement variants of these two interprocessor communication actors using the Message Passing Interface (MPI) (Gropp et al., 1999).

The Message Passing Interface (MPI) is a standardized and portable message-passing system designed for use on parallel computing architectures. MPI is preferred over Pthreads and OpenMP libraries in our implementation because it offers comparable parallelization performance while providing greater flexibility and ease of use. Moreover, MPI supports parallelization across separate devices without requiring shared memory, enabling us to distribute training and inference subsystems across different physical machines. For example, the training process can be run on a computationally intensive machine, while inference can be performed on a more portable embedded device with lower computational power.

The Dataflow Schedule Graph (DSG) provides a graphical representation of schedules based on dataflow semantics. In Wu et al. (2011), the *snd* and *rec* actors are defined as communication actors used to facilitate interaction between parallelized dataflow graphs. As illustrated in Figure 5, the *snd* actor receives tokens from a single input FIFO, *In*_*local*, which collects tokens from the local processor on which the *snd* actor is running. The actor has two output FIFOs: *out*_*local*, which sends tokens back to the local processor, and *out*_*remote*, which handles inter-processor communication by sending tokens to remote processors. The *rec* actor receives tokens from two input FIFOs. The first, *In*_*local*, transfers tokens from the local processor. The second, *In*_*remote*, is used for inter-processor communication, specifically for receiving tokens from other processors. The *rec* actor has a single output FIFO, *out*_*local*, which delivers tokens to other actors running on the same local processor.

**Figure 5:**
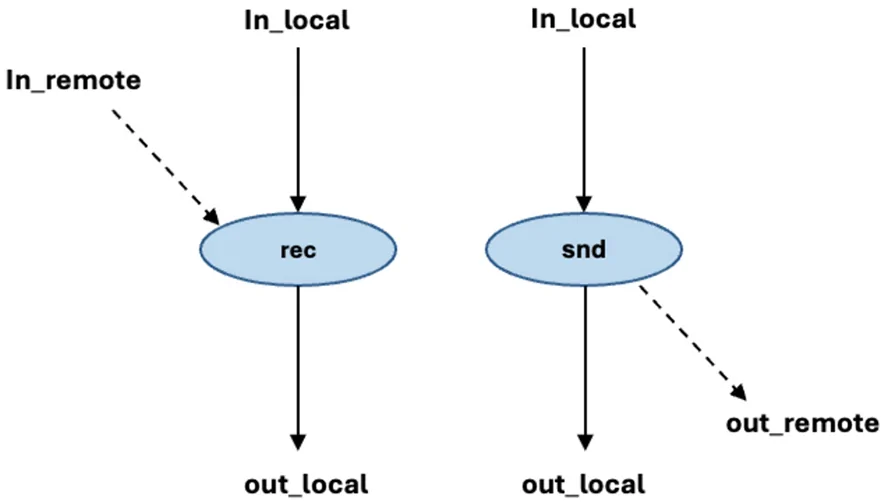
General structure of DSG-based receive (*rec*) and send (*snd*) actors. Solid arrows denote local FIFO communication within a processor group, and dashed arrows denote remote communication between processor groups. The *rec* actor routes local or remote input tokens into the local graph, while the *snd* actor routes local tokens either locally or to a remote graph. RNDC-Lab implements these actors using MPI to coordinate the inference and training graphs and support parallel model updating during real-time inference.

In our implementation, as shown in Figure 4, we implement *snd* and *rec* actors with the MPI library. We also add a *forward* functionality inside the *snd*_*dsg*_*mpi* and *rec*_*dsg*_*mpi* actors, which can be viewed as a function within the associated actor *A* that simply forwards tokens from an input FIFO associated with *A* to an output FIFO. In the *snd*_*dsg*_*mpi* actor, a *send* function takes tokens as a single batch of accumulated samples and sends the batch to other remote processors. On the other hand, in the *rec*_*dsg*_*mpi* actor, a *receive* function receives a *model*_*ready* token, which indicates that a newly trained/updated model is ready from a remote processor and forwards this information to the output FIFO *FIFO*_5_1. The *forward* functions within *snd*_*dsg*_*mpi* and *rec*_*dsg*_*mpi* forward the single neuron signal token from their input FIFO to their output FIFO.

In *Train*_*Graph*, shown in Figure 4, the *activate* actor ensures that the entire *Train*_*Graph* remains actively listening for communication from the *Inference*_*Graph*. It disables listening only when all actors within the *Inference*_*Graph* become inactive. The *rec*_*dsg*_*mpi* actor forwards an activation token generated by the *activate* actor. The internal *rec* actor receives a token containing a batch of accumulated samples and forwards it to the *MP*_*NeuroGRS* actor. The *MP*_*NeuroGRS* actor performs compact structure pruning and outputs the resulting compact DNN model to the *synthesis* actor via FIFO *FIFO*_9. The *synthesis* actor then translates the compact model into a LIDE dataflow application graph and sends a *model*_*ready* token to the *snd* actor through FIFO *FIFO*_10. The *snd* actor, positioned after the *synthesis* actor, waits for the *model*_*ready* token and transmits the signal to remote processors in *Processor*_*group*_1.

The proposed RNDC-Lab framework is a general framework for real-time calcium imaging-based neural decoding systems. Alternative methods and algorithms, such as those resulting from future research, can be readily integrated by wrapping them within the *RNDAE*, *model*_*prepare*, and *model*_*inference* actors. This flexibility and extensibility is provided by the precise and efficient, dataflow-based interfacing constraints provided by LIDE and DSG modeling.

## 4 Experiments

Because the preprocessing and compact-decoder subsystems have already been benchmarked independently in prior studies (Lee et al., 2020; Wu et al., 2021), the experiments in this work focus on the new system-level capabilities of RNDC-Lab, including modular end-to-end integration, concurrent model preparation and inference, and dynamic model replacement.

### 4.1 Datasets

We evaluate RNDC-Lab using both simulated and real-world datasets. The simulated data is carefully designed to test various properties of our proposed framework and system.

#### 4.1.1 Brain Phantom Dataset

To help evaluate the RNDC-Lab framework, we develop the Brain Phantom Dataset, which consists of calcium imaging captures acquired from an *in vitro* mouse-brain phantom that replicates both the gross anatomy and the optical properties of the cortical tissue. The phantom is cast in a 3D printed mold generated from the Allen Brain Atlas (10μm resolution), producing a geometry-accurate mouse brain down to the primary visual cortex (V1) region.

##### 4.1.1.1 Tissue-mimicking matrix

To match the scattering coefficient of gray matter, we pour a 0.7% (w / v) agarose solution doped with reconstituted powdered milk (3.2g mL^−1^) into the mold while the mixture is kept at 75°C. This milk-agarose composite provides a reduced scattering coefficient close to that of cortical tissue in the range470520nm band used by the Miniscope Aharoni and Hoogland (2019), which is the device used for data capture that is used in developing this dataset.

##### 4.1.1.2 Fluorescent “neurons”

Fluorescent polystyrene microspheres are suspended in 0.1% Tween 20 and serially diluted to a target concentration of 50beads/μl. Immediately before gelation, 0.5μl of this suspension is pipetted onto the semi-solid surface, producing *m* 30 discrete beads that serve as neuron surrogates. Each cluster occupies 10–20 pixels (range1530μm) in the recorded field of view, closely approximating the apparent soma size of neurons in the V1 region.

##### 4.1.1.3 Imaging protocol and labels

Data captures utilize a Miniscope–v4 at 30 Hz, 752 × 480-pixel frames acquired while the excitation LED is *on* form the “active” class; those with the LED *off* form the “inactive” class. Accordingly, the behavioral label for frame *f* is


$$y_{f}=\{\begin{array}{cc} 1, & \text{LED on}\\ 0, & \text{LED off} \end{array}$$

which is equivalent to thresholding the mean frame fluorescence. Preliminary imaging confirms that the phantom yields bead-like regions of interest (ROIs) and realistic background texture. However, the captures are noticeably cleaner than typical *in vivo* recordings. Upcoming iterations will boost background noise and fine-tune the neuron-to-background contrast to better match real calcium-imaging data.

#### 4.1.2 N80 dataset

We generate the N80 dataset, which includes distribution shifts to evaluate the effectiveness of the model updating functionality in the RNDC-Lab framework. The N80 dataset consists of five days of simulated neural activity of 80 neurons, with each day comprising 600 simulated frames. The neuron model used in the simulated data is the integrate-and-fire model with additive noise (Gütig and Sompolinsky, 2006; Wu et al., 2020). The neuron model can be represented by the following equation:


$$\begin{array}{l} \frac{dV}{dt}=\frac{V_{\text{rest}}-V}{\tau }+\sigma \times \tau^{\left(-0.5\right)}\times \varepsilon , \end{array}$$ (1)

where *V* represents the membrane potential, *V*_*rest*_ indicates the resting potential, σ represents a parameter that controls the noise, τ is the membrane time constant, and ε represents a Gaussian random variable with mean 0 and standard deviation 1. The membrane potential *V* changes in response to spikes received through synapses. Whenever *V* exceeds a threshold, the neuron model generates a spike. We set the threshold as 1. Each synaptic input from other neurons increases the membrane potential by a weight *w*. A brief period is required between two consecutive spikes from the same neuron, representing the refractoriness of the neuron model.

The 80 neurons are divided into two groups, A and B. The 40 neurons in group A are activated directly by a stimulus, while the 40 neurons in group B receive synaptic inputs from two or three neurons in group A. This dataset simulates a 5-day learning process. The synaptic weights *w* between a neuron in group A and a neuron in group B are set to 0.4, 0.5, 0.6, 0.7, and 0.8 for days 1 through 5, respectively. Higher synaptic weights *w* result in increased activity among neurons in group B. This models a learning process in which a presynaptic spike is followed by a postsynaptic spike, and the synapse between a neuron in group A and a corresponding neuron in group B is progressively strengthened over time. The behavior label for the N80 dataset (i.e., for a given image frame) represents whether the average spike rate of group B is greater than 0.1.

To make the data distribution of each day different from others, we introduce random noise in behavior labels of the dataset of each day by randomly toggling *FR*×600 amount of binary behavior labels. *FR* is called the flip rate. For the N80 dataset, we also introduce noise into the simulated calcium imaging frames using a noise level of η = 0.1. Motion and rotation artifacts are not introduced in the simulation.

#### 4.1.3 Reward learning dataset

A simulated dataset of a reward learning task was generated to evaluate multiclass classification performance. In this task, mice are trained on two reward-predicting odors: odor A, associated with a high reward, and odor B, associated with a low reward. The simulation consists of five sessions, with each session containing five trials for odor A and five trials for odor B. The trial timing is structured as follows: a 2-second pre-odor period, odor presentation at the 2-second mark lasting for 4 seconds, and reward delivery at 7.5 seconds lasting for 0.1 seconds. Our simulation models the post-learning phase of the task, after the odor-reward association has been fully established.

The simulation includes 30 neurons randomly distributed across a region, modeled using an integrate-and-fire model with additive noise. Because the simulation models the phase after conditioned response has been established, it does not include synaptic adaptation. Action potentials are evoked by odor presentation, where the differing reward magnitudes are modeled via the injected stimulus current. For the low-reward condition (odor B), the injected stimulus current is set to 0.45, a parameter chosen to generate a medium level of firing. For the high-reward condition (odor A), the injected stimulus current is set to 0.50, 0.55, or 0.60. We varied the stimulus current for the high-reward condition to assess its impact on classification performance, ensuring that the resulting firing probability for odor A is always greater than that for odor B.

Simulated motion artifacts are as follows. For incremental motion, the standard deviations for x-translation, y-translation, and rotation are 0.05 pixels, 0.05 pixels, and 0.0012 radians, respectively. The correlations between x-translation and y-translation, x-translation and rotation, and y-translation and rotation are 0, 0.01, and -0.01, respectively. The motion patterns across the three datasets (representing the varying stimulus currents for the high-reward condition) are identical. Because the simulated imaging field of view (FOV) is restricted, only 14 of the 30 neurons are observable. This reflects that calcium imaging typically captures only a subset of the neurons involved in a cognitive process. The imaging sampling rate is 10 Hz, resulting in 100-ms frames.

Pupillometry serves as a behavioral proxy for attention and task engagement. In this simulation, pupil diameter is assumed to vary directly with population neural activity. The underlying ground-truth brain state is defined by the average population firing rate of all 30 neurons, which is then discretized into three categories: a baseline state, a low engagement state, and a high engagement state. The discretization process is as follows. Because the population firing rate for no-odor frames is close to zero, we use a very small value (0.01) to threshold population rates into zero or non-zero. For non-zero values, we use the sample median to discretize them into low or high. The baseline state corresponds to a population rate close to zero, the low-engagement state to a population rate below the sample median of non-zero samples, and the high-engagement state to a population rate above the sample median of non-zero samples. We chose this algorithm because the population rate is zero-inflated (due to the absence of a cue), and the non-zero portion demonstrates a bimodal pattern. This categorical pupillometry readout reflects these underlying engagement states. Therefore, cue presentation and cue type (which reflects reward magnitude) elicit population neural activity. This neural activity defines a brain state, which in turn determines the categorical pupil diameter variable used as the framewise label.

#### 4.1.4 N90 dataset

In this work, we introduce the N90 dataset to facilitate experimentation with RNDC-Lab on preprocessing. This dataset comprises 90 neurons simulated using the neuron model described in Equation 1. These 90 neurons are divided into three groups of 30 neurons each. Neurons in groups 1 and 2 are simulated to be activated by external stimuli, whereas neurons in group 3 respond to synaptic inputs from four upstream neurons (two from group 1 and two from group 2). The behavior label for a given image frame is set to “1” if three or more neurons in group 3 fire, and “0” otherwise.

For the simulated calcium imaging frames, we use neuron masks from the neurofinder00 database (Pnevmatikakis et al., 2016). For each of the 1200 frames per simulated dataset, we set the pixel intensities within an active neuron's mask to λ = 128; for inactive neurons, we set the intensities to λ = 0. We simulated the calcium reporter *jGCaMP*7*F*, which has a decay half-life of approximately 265ms (Dana et al., 2019) (corresponding to roughly eight simulated frames at an acquisition rate of 30Hz).

We introduce three control parameters–η, α, and β–to generate simulated frames with noise, motion, and rotation, respectively. Motion is simulated by randomly shifting the frame centroid in the *x* or *y* direction by a value drawn from a uniform distribution in [−α, α]. The translation parameter α is measured in pixels. The rotation angle about the frame center is randomly sampled from [0, β]. A similar calcium imaging simulation strategy was also employed in Lee et al. (2020). Finally, to incorporate noise at a level η, we add values drawn from a Gaussian distribution with a mean of 0 and a standard deviation of σ = η × λ to each pixel intensity.

#### 4.1.5 Medium spiny neurons

An existing real-world calcium imaging dataset (Barbera et al., 2016) of medium spiny neurons (MSNs) is used to assess RNDC-Lab adaptation. A customized miniature microscope was used to image the calcium fluorescence of D1 MSNs in the dorsal striatum. Calcium imaging data were obtained during an open-field experiment to assess mouse locomotion. We placed the mice in an open field, acquired GCaMP6s fluorescence from the dorsal striatum through the miniscope, and concurrently recorded mouse locomotor activity using a video-recording system. On the day of recording, we acquired three 5-minute sessions of simultaneous recordings of the GCaMP6s fluorescent signal and mouse behavior. A 5-minute resting period was incorporated between consecutive sessions. Each session consisted of 3,000 frames at a frame rate of 10 Hz. The behavioral variable of interest was ambulation, defined as any period when the animal's velocity exceeded 3 cm/s for at least 0.5 seconds, which was treated as a binary variable. The 0.5 s constraint was enforced computationally by the run-length algorithm.

The MSN data used in this work were preprocessed before being provided to the RNDC-Lab decoding subsystem. The preprocessing includes motion correction, cell detection, and neural feature extraction. These operations are similar to the corresponding preprocessing stages implemented in the RNDC-Lab NDSEP subsystem. The resulting neural traces and frame-aligned behavioral labels are used as inputs to the preprocessed-data pipeline.

### 4.2 Systems evaluated in experiments

The proposed system is the integrated RNDC-Lab system with NDSEP, NeuroGRS, NGSynth, as presented in Section 3. The first baseline approach combines NDSEP, NeuroGRS, and NGSynth without using the RNDC-Lab framework or the dynamic loading and dataflow communication actors *snd* and *rec*. We refer to this as the *runtime baseline*. This baseline relies on Bash scripts to connect the NDSEP, NeuroGRS, and NGSynth subsystems. The combined system cannot run training and inference in parallel; therefore, it cannot re-train a new decoding model with NeuroGRS and replace the existing model while inference is ongoing. Additionally, data transfer between NDSEP and NGSynth occurs via files rather than FIFOs within a single dataflow application graph. Specifically, signals generated by NDSEP are first saved to a file to disk and then loaded by NGSynth for inference. As a result, this baseline approach incurs higher runtime overhead for communication between the separate dataflow graphs of NDSEP and NGSynth.

The second comparison baseline disables the model updating functionality of the RNDC-Lab framework. In this baseline, the classifier is trained only once using the first batch of samples collected by the *signal*_*accumulator* and is then used to classify the incoming data stream. We refer to this as the *static mode*. This mode is used to compare against the system with the model updating functionality enabled. The comparison primarily evaluates decoding model accuracy on data streams with non-stationary distributions. It also allows assessment of the runtime differences in real-time inference with and without model updating.

The runtime baseline and the static baseline are included in experiments comparing our proposed system and framework to demonstrate the following: (1) the proposed system with the RNDC-Lab framework can achieve real-time, end-to-end neural decoding for calcium imaging; (2) the model updating mechanism of the RNDC-Lab framework is effective for concept drift; and (3) each functional unit of the proposed system operates correctly.

### 4.3 Motion correction experiments

The motion correction subsystem is a critical stage for obtaining reliable neural signals. Our system performs Euclidean motion correction using the enhanced correlation coefficient (ECC) algorithm. To estimate motion shifts, the algorithm requires a reference image. We evaluated three reference-image strategies: (1) using the first frame as the reference, (2) using a static mean image computed from a fixed number of initial frames, and (3) using a dynamic mean image that is updated incrementally as additional frames are processed.

For the static mean strategy, the reference image is computed as the mean of the first *n* frames, where *n* is a hyperparameter, and is then fixed for all subsequent motion correction. The first *n* frames must be stored in memory until the reference image has been computed, after which these frames are processed using the computed reference image. This results in additional memory usage and processing time. In contrast, the dynamic mean strategy updates the reference image incrementally as each frame is processed. Specifically, after processing of the first frame is complete, the first frame is used as the reference image. After processing the second frame, the reference image is updated as the mean of the first two frames. Similarly, after processing the third frame, the reference image is updated as the mean of the first three frames, and this process continues for subsequent frames. Thus, at frame *t*, the dynamic reference image is computed as


$$\begin{array}{l} R_{t}=\frac{S_{t}}{t},\;S_{t}=S_{t-1}+I_{t}, \end{array}$$ (2)

where *I*_*t*_ denotes the motion-corrected image at frame *t*, *S*_*t*_ denotes the cumulative sum of the motion-corrected images up to frame *t*, and *R*_*t*_ denotes the dynamic reference image at frame *t*. The cumulative mean is maintained using the cumulative sum and the number of processed frames, allowing the reference image to be updated without storing previously processed frames or recomputing the mean over all frames. Consequently, the dynamic strategy incurs low computational and memory overhead for reference-image updates.

The dynamic strategy is intended to provide a reference image that progressively improves the representation of the observed data as additional frames are processed. Importantly, the dynamic reference image is computed from motion-corrected frames, so the frames contributing to the reference are maintained in a common spatial coordinate system. This reduces the potential for changes in the reference image to arise solely from frame-to-frame motion. However, because new frames are continuously incorporated into the cumulative mean, the reference image can gradually change over time. Such changes represent a potential failure mode if substantial changes in the underlying image content occur over time. The experimental results below evaluate the effect of different numbers of frames used in the reference-image computation.

Table 1 presents the mean balanced accuracy and mean runtime of the NDSEP actor using the Brain Phantom dataset. For each reference-image configuration, the evaluation considers the first *n* frames containing at least one neuron, with *n* ranging from 100 to 600 for the dynamic mean strategy. Balanced accuracy is calculated from the neural-signal classification results by averaging the recall across the individual classes, thereby accounting for class imbalance. When using a dynamic mean of 100 frames instead of the static mean, the runtime is reduced by more than 4ms while achieving comparable balanced accuracy. For the dynamic mean of 200 frames, the average runtime is noticeably higher than for the other configurations. This is likely because the resulting reference image is less representative of the subsequent frames, causing the ECC algorithm to require more iterations on average to converge. One possible explanation is that the frames incorporated between 100 and 200 frames contain greater accumulated motion, resulting in a less representative mean image. As additional frames are incorporated between 200 and 300 frames, the reference image becomes more representative of the subsequent frames, which can improve convergence. This is reflected in the results, where the runtime for the 300-frame dynamic mean is lower than that for the 200-frame dynamic mean.

**Table 1:** Mean runtime of NDSEP subsystem for 1,000 frames and balanced accuracy using Brain Phantom dataset in static mode.

Reference image type	Frames	Runtime (ms)	Accuracy
first frame	1	26.824	0.500
static mean	100	28.991	0.933
Dynamic mean	100	24.411	0.930
	200	32.287	0.957
	300	24.078	0.960
	400	24.931	0.957
	500	25.667	0.952
	600	25.866	0.950

Moreover, we achieved a maximum of 96% accuracy using a dynamic mean of 238 frames as the reference image. This improvement occurs because the reference frame is better than the other cases, allowing the motion correction algorithm to converge faster and produce more reliable results.

### 4.4 Runtime experiments

We use 1,000 frames from the Brain Phantom Dataset for the runtime experiment. Each frame consists of a 400 × 400 pixel image. All critical functionalities of *RNDAE*_*NDSEP*, including motion correction, are enabled to ensure that the measured runtime reflects the complete operation of the system. We set *MP*_*NeuroGRS* to have an initial model as a multi-layer perceptron with 64 × 32 × 16 shape, i.e., with three hidden fully connected layers having 64, 32, and 16 artificial nodes. The runtime experiment is performed on a desktop computer equipped with an Intel Core i7-14700 processor.

The runtime experiment results for the proposed system are presented in Tables 2 and 3. Using 1,000 frames, we measured the wall-clock execution time immediately before and after invoking each actor in both the inference and training graphs. The experiment was repeated for 10 independent end-to-end runs under both static and dynamic modes. For each run, the maximum, mean, and minimum runtimes were calculated over all invocations of each actor. The values reported in the tables are the averages of the corresponding maximum, mean, and minimum values across the 10 runs. The dynamic mean of 238 frames was used for the experiment since it achieved the highest accuracy.

**Table 2:** Runtime of actors in the inference graph for 1,000 frames.

Actor name	Static mode (ms)			Dynamic mode (ms)		
	Max	Mean	Min	Max	Mean	Min
Signal accumulator	0.028	0.002	0.001	0.041	0.004	0.001
snd_dsg_mpi	0.004	0.000	0.000	0.014	0.002	0.001
Actor grs commu_alldone	0.030	0.000	0.000	0.055	0.000	0.000
MI_NGSynth	6.768	0.087	0.008	6.744	0.118	0.008
RNDAE_NDSEP	65.802	20.449	10.404	67.016	20.789	10.587
rec_dsg_mpi	0.026	0.003	0.001	0.032	0.004	0.001
signal_stabilizer	0.098	0.015	0.006	0.096	0.016	0.006
Sum of all actors	72.755	20.558	10.421	73.996	20.934	10.579

**Table 3:** Runtime of actors in the training graph for 1,000 frames.

Actor name	Static mode (ms)			Dynamic mode (ms)		
	Max	Mean	Min	Max	Mean	Min
MP_NeuroGRS+synthesis	3,417.169	3,417.169	3,417.169	5,368.210	4,549.075	3,439.603
rec_dsg_mpi	0.170	0.170	0.170	0.196	0.185	0.170
snd_dsg_mpi	0.005	0.005	0.005	0.007	0.006	0.004
active	0.336	0.003	0.003	0.687	0.003	0.003
sum of all actors	3,417.681	3,417.348	3,417.348	5,369.108	4,549.268	3,439.748

As shown in Table 2, the inference graph maintains low processing latency under both modes. The summed actor-specific maxima are at 72.755 ms in static mode and 73.996 ms in dynamic mode. The corresponding mean runtimes are 20.558 ms and 20.934 ms, respectively, indicating that dynamic model updating introduces only limited overhead into the inference graph.

Table 3 shows that model synthesis dominates the training graph runtime. In static mode, synthesis is performed only once, yielding approximately 3.42s. In dynamic mode, repeated model updates use increasing amounts of accumulated training data, resulting in synthesis runtimes ranging from approximately 3.44s to 5.37s, with a mean of 4.55s.

Importantly, RNDC-Lab executes the training and inference graphs concurrently. While a new model is being synthesized in the training graph, the inference graph continues decoding incoming frames using the most recently available model. Once the updated model becomes available, the inference graph switches to the new model for subsequent inference. This design allows RNDC-Lab to perform model updating without stalling real-time inference while still incorporating newly available data into model updates for subsequent decoding.

Before signal stabilization, the mean per-frame preprocessing latency is 22.59 ms in static mode and 23.19 ms in dynamic mode. These values are reported as a reference for the initialization stage of the complete pipeline. In practical streaming applications, part of this startup work, including initial model preparation, can be performed before online decoding begins, for example by loading a pretrained initial model.

To further evaluate runtime performance from a streaming-system perspective, we measure the per-frame end-to-end (E2E) latency. Unlike the actor-level measurements above, the E2E latency captures the elapsed time from the start of processing an input frame to its completion in the pipeline. For the 297 prediction frames after signal stabilization, Table 4 summarizes the E2E prediction latency, and Figure 6 shows the corresponding latency distributions for the static and dynamic modes.

**Table 4:** Per-frame end-to-end prediction latency after signal stabilization.

Measurement	Static mode	Dynamic mode
Mean (ms)	58.86	81.93
Minimum (ms)	18.79	18.83
Maximum (ms)	130.60	135.82
Median (ms)	57.49	78.94
95th percentile (ms)	78.19	123.46
99th percentile (ms)	98.60	132.11
Prediction frames >100 ms	2/297 (0.67%)	57/297 (19.19%)

**Figure 6:**
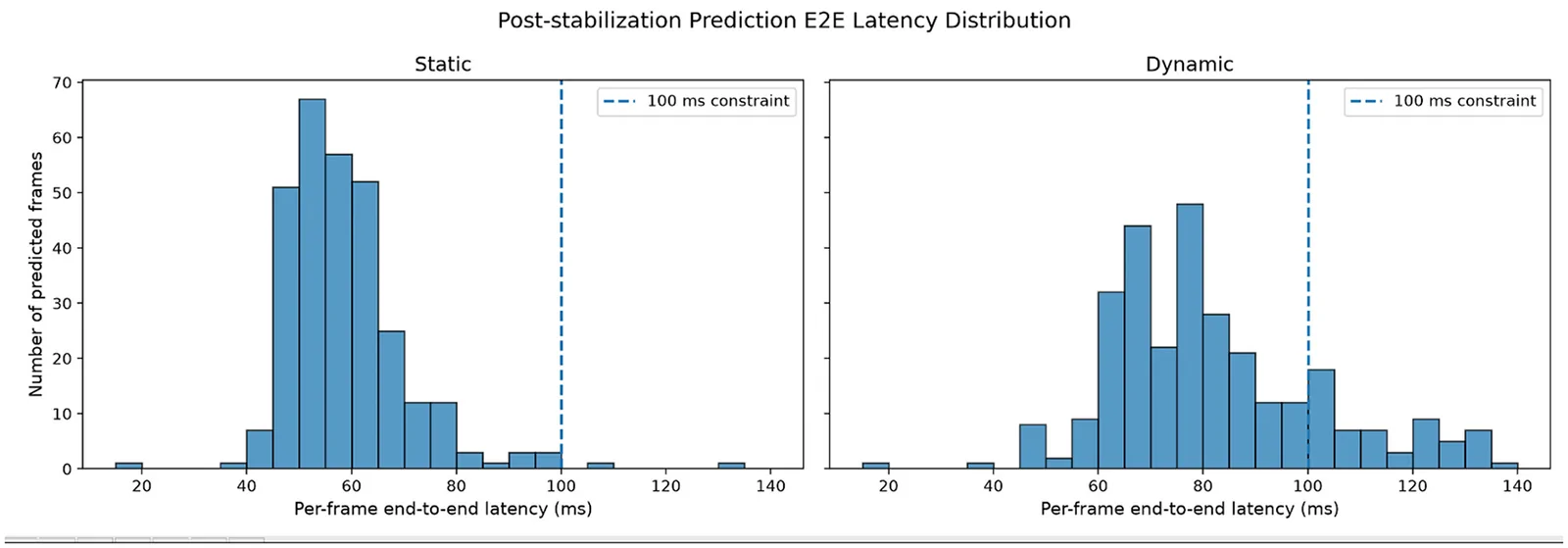
Distribution of per-frame E2E prediction latency after signal stabilization for the static and dynamic modes.

We use 100 ms as the per-frame real-time constraint, corresponding to the target decoding frequency of 10 Hz. The static mode achieves a mean E2E prediction latency of 58.86 ms, with 2 out of 297 prediction frames (0.67%) exceeding the 100 ms constraint. The dynamic mode has a higher mean latency of 81.93 ms, with 57 out of 297 prediction frames (19.19%) exceeding 100 ms. The percentages of frames exceeding 100 ms are calculated over the frames that produce decoding outputs after signal stabilization. The distribution also shows a wider high-latency tail in the dynamic mode, with a 95th percentile of 123.46 ms compared with 78.19 ms in the static mode. This higher E2E latency is expected because dynamic mode performs concurrent model training and model replacement while inference continues, resulting in additional contention for shared processing resources. Nevertheless, the additional mean E2E latency remains only approximately 23 ms compared with static mode.

The actor-level and E2E measurements provide complementary views of runtime performance. Actor-level timing identifies which actors dominate the computation and could be prioritized for optimization, while per-frame E2E timing captures the latency experienced by the complete streaming pipeline. Together, these two levels of analysis provide RNDC-Lab with systematic profiling, performance diagnosis, and optimization capabilities.

### 4.5 Decoding experiments

For the experiments in this section, we primarily focus on decoding performance as measured by the balanced accuracy metric. This choice is motivated by class imbalance in the evaluated datasets. In our context, balanced accuracy provides a more meaningful evaluation than standard accuracy.

#### 4.5.1 Multiclass classification experiments

We evaluated RNDC-Lab performance on three reward-learning datasets (see Section 4.1.3) to determine (1) whether the decoder can distinguish three behavior states (a baseline state, a low engagement state, and a high engagement state) and (2) how separation in reward magnitudes affects the balanced accuracy. In this experiment, reward magnitude was parameterized by the injected current. The high-reward (odor A) injected current was evaluated at three levels (0.50, 0.55, and 0.60), while the low-reward (odor B) injected current was fixed at 0.45. Let *I* denote the injected current of odor A. Classes 0, 1, and 2 corresponded to the baseline, low-engagement, and high-engagement states, respectively. Here, we considered only the dynamic mean reference image, which was found to be more effective than the static mean reference image (see Table 1). The initial training set was fixed at 1,000 samples across all experiments. In static mode, the model was trained using only these initial 1,000 samples; in dynamic mode, an additional 1,000 samples were added at each subsequent update. Once the RNDC-Lab system stabilized and the first model was ready, all remaining frames served as the test set to evaluate the framework's balanced accuracy. The balanced accuracy is evaluated end-to-end across the full system (preprocessing and decoding).

REFERENCE_COUNT specifies the number of frames used to compute the mean reference frame. We varied REFERENCE_COUNT from 100 to 1,000 in increments of 100. Figure 7 shows the balanced accuracy obtained at different REFERENCE_COUNT values for each current level and update mode. The optimal REFERENCE_COUNT varies depending on the injected current. At *I* = 0.60, the highest balanced accuracy (0.740) is achieved when REFERENCE_COUNT=100, whereas at *I* = 0.55, the best performance occurs at REFERENCE_COUNT=400. At *I* = 0.50, the highest accuracy is reached at REFERENCE_COUNT=1000. For *I* = 0.55 and *I* = 0.60, accuracy generally decreases as REFERENCE_COUNT increases beyond 400, although this trend is not strictly monotonic. The static and dynamic modes exhibit similar trends across REFERENCE_COUNT values at all current levels, indicating that the choice of update mode has a limited effect on sensitivity to REFERENCE_COUNT.

**Figure 7:**
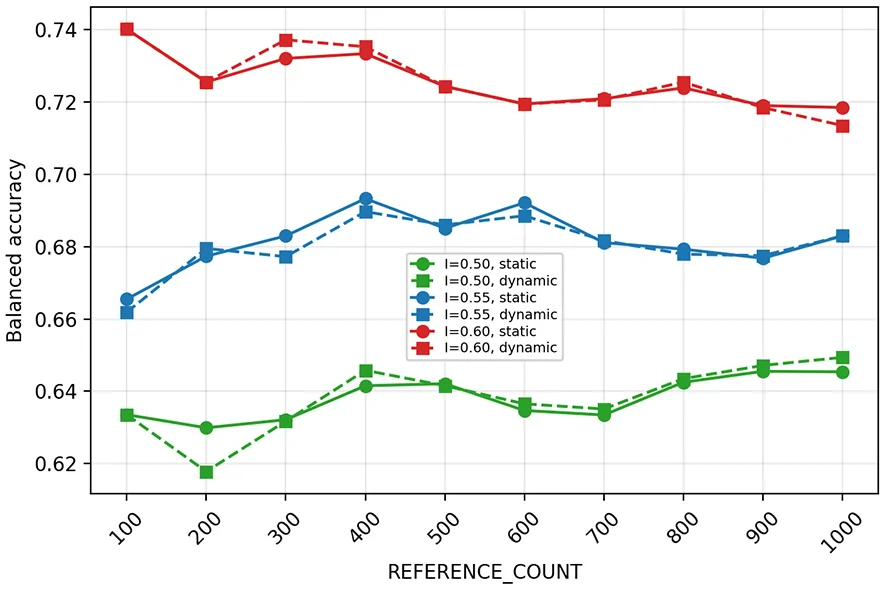
Balanced accuracy across REFERENCE_COUNT values, for each injected current level and update mode.

To ensure a fair comparison across current levels and update modes, we used a single REFERENCE_COUNT value for all conditions. We fixed REFERENCE_COUNT at 400, which was identified as the generally optimal value from our earlier analysis. Beyond this threshold, balanced accuracy tended to degrade. The resulting balanced accuracies for this fixed configuration are presented in Table 5. As the injected current increases, the balanced accuracy also increases, ranging from 0.6415–0.6458 at *I* = 0.50 to 0.7333–0.7352 at *I* = 0.60. Overall, the dynamic mode slightly outperforms the static mode at *I* = 0.50 and *I* = 0.60. However, the performance difference between the two modes is marginal. This similarity is expected, as there is no concept drift in the data.

**Table 5:** Balanced accuracy at the single overall-optimal configuration (REFERENCE_COUNT=400) across all injected current levels and model updating modes.

Current	Mode	Balanced accuracy
*I* = 0.50	Static	0.6415
*I* = 0.50	Dynamic	0.6458
*I* = 0.55	Static	0.6933
*I* = 0.55	Dynamic	0.6897
*I* = 0.60	Static	0.7333
*I* = 0.60	Dynamic	0.7352

As *I* increases, the separation in reward magnitudes also increases. As shown in Table 5, balanced accuracy increases with *I* for both the static and dynamic modes. Therefore, decoding capability improves with greater separation in reward magnitudes.

Table 6 presents the confusion matrix for the configuration at *I* = 0.60 in dynamic mode, yielding a balanced accuracy of 0.7352. Class 0 achieves the highest recall of 0.973, followed by Class 2 with a recall of 0.767, while Class 1 exhibits the lowest recall of 0.466. The majority of classification errors involve Class 1 being assigned to Class 0, with 320 out of 764 true Class 1 samples misclassified as Class 0, compared to 88 samples misclassified as Class 2. In contrast, confusion between Classes 0 and 2 is limited, with only 13 Class 0 samples misclassified as Class 2 and 23 Class 2 samples misclassified as Class 0. Overall, these results indicate that RNDC-Lab achieves reasonable decoding performance.

**Table 6:** Confusion matrix for *I*=0.60 in dynamic mode (balanced accuracy = 0.7352).

	Prediction = 0	Prediction = 1	Prediction = 2
Actual = 0	1,993	43	13
Actual = 1	320	356	88
Actual = 2	23	117	461

#### 4.5.2 Dynamic mode for distribution shifts

The distribution-shift simulation in this section is related to representational drift, where neural activity patterns associated with task-relevant variables can change over time (Driscoll et al., 2017; Ratzon et al., 2024). To provide a controlled system-level abstraction of this challenge, we compare the static baseline with the proposed RNDC-Lab system to evaluate whether online model updating can maintain decoding performance when the input-output relationship changes over time. We use the N80 dataset described in Section 4.1.2 for this experiment. The experiment involves a sequence of 3,000 frames, composed of five different subsets of 600 frames each. We refer to these subsets as *day*_1_, *day*_2_, *day*_3_, *day*_4_, and *day*_5_. We simulate two variations by applying different flip rate (*FR*) settings to each day's dataset. In variation 1, the flip rates are assigned as follows: *day*_1_ receives *FR* = 0.2, *day*_2_ receives *FR* = 0.15, *day*_3_ receives *FR* = 0.1, *day*_4_ receives *FR* = 0.05, and *day*_5_ receives *FR* = 0. In variation 2, only *day*_1_ receives label noise with *FR* = 0.2, while all other days have *FR* = 0. Compared to variation 2, variation 1 introduces more pronounced distributional shifts across the dataset, making it a more challenging scenario for evaluating model adaptability.

For the balanced accuracy results in this subsection, we report *mean*±*std* over ten repeated experiments, where *std* represents the standard deviation. The same simulated data streams are used across the ten runs, while the decoding model is independently trained in each run. Minor performance differences may therefore arise from variations in the training process. Thus, the reported variation reflects differences in model quality across the ten runs.

Before using these simulated datasets to evaluate the effectiveness of the model updating functionality, we first assess two conditions: (1) each dataset is individually learnable, and (2) the distribution of *day*_1_ differs from those of the other datasets. For this verification, we use ground-truth models (*GTM*s), which operate directly on the ground-truth neural activity. We first use a multi-layer perceptron with shape 64 × 32 × 16 as the *GTM*-*MLP*, implemented in Python using TensorFlow. To test learnability, we train the *GTM*-*MLP* on the first 150 samples and test it on the remaining 450 samples within each dataset; we refer to this as the *self-trained* approach. To evaluate distributional difference, we train the *GTM*-*MLP* on the first 150 samples of *day*_1_ and test it on the last 450 samples of each day; we refer to this as the *cross-trained* approach. Using the same cross-trained protocol, we also evaluate logistic regression (*GTM*-*LR*) and support vector machine (*GTM*-*SVM*) decoders as linear and nonlinear non-DNN references, respectively. These reference models are trained once and are not updated during evaluation. Under the fixed data split and settings, *GTM*-*LR* and *GTM*-*SVM* are deterministic and produce identical results across repeated runs, while the *GTM*-*MLP* shows small variation due to stochastic training and random weight initialization.

The verification results for the two variations of the N80 dataset are presented in Tables 7, 8. The identical *day*_5 cross-trained *GTM*-*MLP* values in both tables are intentional: both variations use the same *day*_1 training subset and a *day*_5 test set with no label noise, allowing us to highlight how gradual vs. sudden label-noise changes in the earlier days affect generalization. The balanced accuracies of the self-trained *GTM*-*MLP* for all datasets exceed 50%, indicating that each dataset contains sufficient information to be modeled effectively. The balanced accuracies of the cross-trained *GTM*-*MLP*—trained on *day*_1 and tested on *day*_2 through *day*_5—are lower than those achieved by the corresponding self-trained models, suggesting that the distribution of *day*_1 differs from the later-day datasets. The LR and SVM decoders show similar cross-day trends, providing additional evidence that the observed performance degradation is associated with the simulated distribution shift rather than being specific to the MLP decoder. We retain the MLP as the decoding model in the current RNDC-Lab implementation because its nonlinear architecture provides flexibility for online model updating and for future architecture optimization and model-compression techniques under real-time constraints. In summary, we have verified that these two sets of simulated calcium imaging frames are suitable for testing the effectiveness of the model updating functionality in our proposed system.

**Table 7:** Balanced accuracy results for verifying N80 dataset Variation 1 using different decoders.

	*day*_1 (flipRate=0.2)	*day*_2 (flipRate=0.15)	*day*_3 (flipRate=0.1)	*day*_4 (flipRate=0.05)	*day*_5 (flipRate=0)
Self-trained *GTM*-*MLP*	0.639 ± 0.013	0.719 ± 0.007	0.832 ± 0.008	0.920 ± 0.008	0.996 ± 0.001
Cross-trained *GTM*-*MLP*	0.639 ± 0.013	0.653 ± 0.012	0.741 ± 0.008	0.816 ± 0.014	0.871 ± 0.019
Cross-trained *GTM*-*LR*	0.665	0.706	0.795	0.857	0.947
Cross-trained *GTM*-*SVM*	0.607	0.667	0.742	0.777	0.835

**Table 8:** Balanced accuracy results for verifying N80 dataset Variation 2 using different decoders.

	*day*_1 (flipRate=0.2)	*day*_2 (flipRate=0)	*day*_3 (flipRate=0)	*day*_4 (flipRate=0)	*day*_5 (flipRate=0)
Self-trained *GTM*-*MLP*	0.639 ± 0.013	0.980 ± 0.002	0.988 ± 0.002	0.994 ± 0	0.996 ± 0.001
Cross-trained *GTM*-*MLP*	0.639 ± 0.013	0.803 ± 0.015	0.861 ± 0.014	0.868 ± 0.012	0.871 ± 0.019
Cross-trained *GTM*-*LR*	0.665	0.880	0.942	0.923	0.947
Cross-trained *GTM*-*SVM*	0.607	0.823	0.847	0.829	0.835

After verifying that the simulated datasets are both informative and exhibit differences from one another, we designed experiments to demonstrate the effectiveness of the model updating functionality in our proposed system. In these experiments, we refer to the static baseline described in Section 4.2 as the *static*_*mode*, and our proposed system as the *dynamic*_*mode*. The *static*_*mode* has its model updating functionality disabled, while the *dynamic*_*mode* has it enabled. All other configurations are identical between the two modes. More specifically, the *static*_*mode* trains a single classifier using only the first collected batch of neuron signals and does not update the model with subsequent calcium imaging frames. In contrast, the *dynamic*_*mode* continuously trains new models and replaces the previous one whenever a new batch of samples is collected from the incoming data stream. The decoding performance metrics generated by the *dynamic*_*mode* are expected to be higher than those of the *static*_*mode*, since the *dynamic*_*mode* can update the decoding model in parallel with the inference cycle. This improvement will demonstrate the effectiveness of the model updating mechanism built into the RNDC-Lab framework.

We run both the *static*_*mode* and *dynamic*_*mode* on Variations 1 and 2 of the N80 dataset. For the *dynamic*_*mode*, we configure different update rates to study the relationship between update frequency and overall decoding performance. The *static*_*mode* is trained once using the first 150 samples from *day*_1_. We denote a model update occurring after every *N* frames as *updateRate* = *N* frames.

As visualized in Figure 8 and reported in Tables 9, 10, the balanced accuracies of the *dynamic*_*mode* are higher than those of the *static*_*mode* across both variations of the N80 dataset and for each individual day subset—except for *day*_1_. This exception is expected, as both modes are initially trained on data from *day*_1_. When comparing Variations 1 and 2, the *static*_*mode* exhibits similar performance in both cases, indicating its inability to adapt to distributional changes. In contrast, the *dynamic*_*mode* shows higher performance in Variation 2, highlighting the effectiveness of its model updating capability in adapting to less frequent distribution shifts. This performance difference can be attributed to two main reasons. First, the calcium imaging frames from *day*_1_ are too noisy to provide the model with reliable information for accurately predicting future data, limiting the effectiveness of the *static*_*mode*. Second, in Variation 2, the datasets from *day*_2_, *day*_3_, and *day*_4_ contain no label noise, which allows the *dynamic*_*mode* not only to achieve higher balanced accuracy but also to incrementally train more accurate models as new clean data arrives.

**Figure 8:**
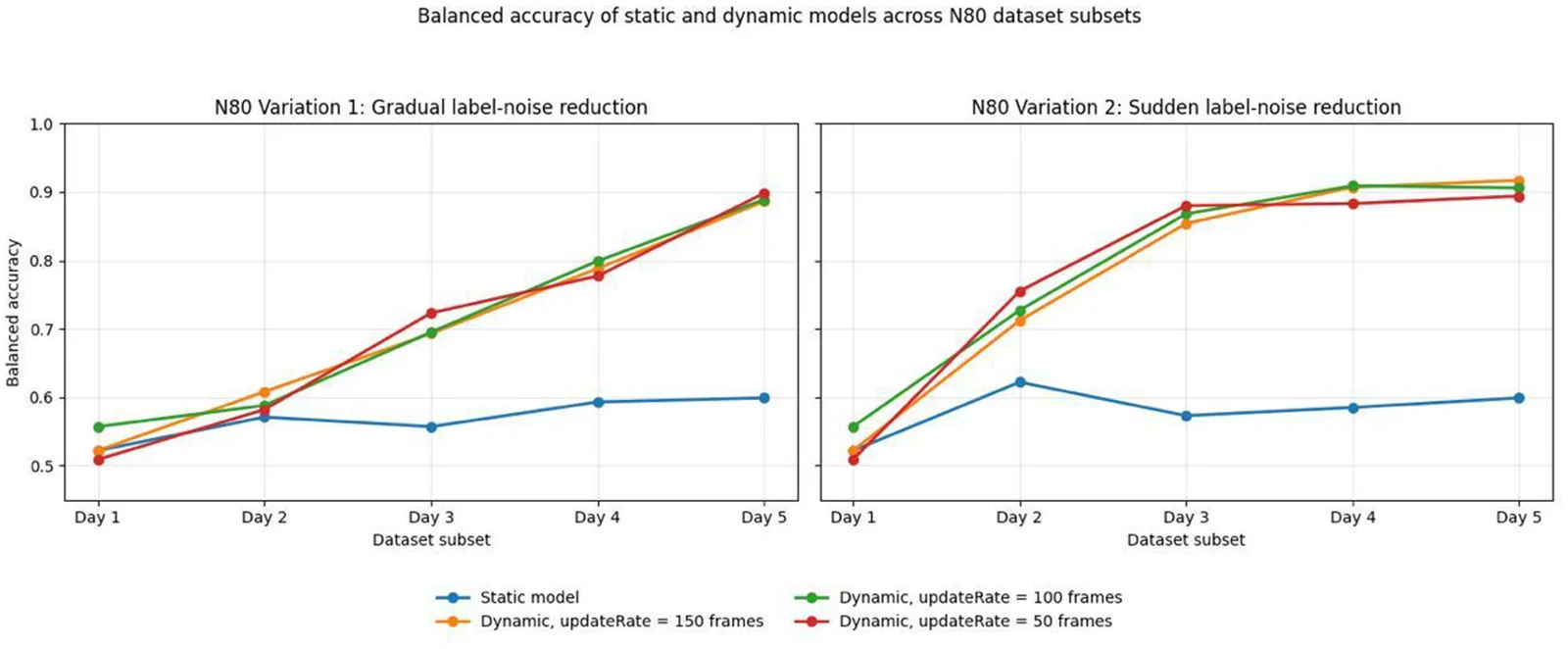
Balanced accuracy in static and dynamic modes across the N80 dataset subsets. The dynamic mode generally outperforms the static mode after *day*_1_, demonstrating the benefit of online model updating under gradual and sudden label-noise reduction settings.

**Table 9:** Results of neural decoding with *static*_*mode* and *dynamic*_*mode* with different updating rates on Variation 1 of the N80 dataset.

	*static*_*mode*	*dynamic*_*mode* (updateRate = 150 frames)	*dynamic*_*mode* (updateRate = 100 frames)	*dynamic*_*mode* (updateRate = 50 frames)
*day*_1_(flipRate=0.2)	0.522 ± 0.022	0.522 ± 0.022	0.557 ± 0.06	0.509 ± 0.023
*day*_2_(flipRate=0.15)	0.571 ± 0.012	0.608 ± 0.009	0.588 ± 0.01	0.582 ± 0.017
*day*_3_(flipRate=0.1)	0.557 ± 0.024	0.693 ± 0.018	0.695 ± 0.013	0.723 ± 0.012
*day*_4_(flipRate=0.05)	0.593 ± 0.022	0.788 ± 0.013	0.799 ± 0.012	0.777 ± 0.009
*day*_5_(flipRate=0)	0.599 ± 0.027	0.886 ± 0.007	0.889 ± 0.009	0.898 ± 0.007
*day*_1_ to *day*_5_	0.577 ± 0.017	0.730 ± 0.007	0.730 ± 0.005	0.730 ± 0.007

**Table 10:** Results of neural decoding with *static*_*mode* and *dynamic*_*mode* with different updating rates on Variation 2 of the N80 dataset.

	*static*_*mode*	*dynamic*_*mode* (updateRate = 150 frames)	*dynamic*_*mode* (updateRate = 100 frames)	*dynamic*_*mode* (updateRate = 50 frames)
*day*_1_(flipRate=0.2)	0.522 ± 0.022	0.522 ± 0.022	0.557 ± 0.06	0.509 ± 0.023
*day*_2_(flipRate=0)	0.622 ± 0.028	0.712 ± 0.014	0.727 ± 0.022	0.755 ± 0.012
*day*_3_(flipRate=0)	0.573 ± 0.024	0.854 ± 0.012	0.868 ± 0.011	0.880 ± 0.005
*day*_4_(flipRate=0)	0.585 ± 0.019	0.907 ± 0.013	0.909 ± 0.007	0.883 ± 0.015
*day*_5_(flipRate=0)	0.599 ± 0.027	0.917 ± 0.006	0.906 ± 0.007	0.894 ± 0.026
*day*_1_ to *day*_5_	0.589 ± 0.019	0.836 ± 0.006	0.843 ± 0.007	0.841 ± 0.012

As shown in Tables 9, 10, we evaluate the decoding performance in *dynamic*_*mode* in terms of balanced accuracy under different model update rates: *updateRate*∈{50, 100, 150}. This experiment is designed to explore how the update frequency affects performance. We hypothesize that more frequent updates (i.e., lower values of *updateRate*) will lead to improved balanced accuracy. For variation 1 of the N80 dataset, the overall balanced accuracy between *day*_1_ and *day*_5_ remains nearly unchanged at different update rates. In variation 2, we observe a slight improvement in decoding performance when decreasing *updateRate* from 150 to 100. This limited gain is likely due to the fact that the model already reaches near-optimal performance, constrained by the inherent noise in the data set in both the simulated frames and the behavioral labels.

In summary, RNDC-Lab in dynamic mode achieves higher balanced accuracy than the static mode across the later-day datasets, while in several cases approaching the performance of the offline GTM-MLP, LR, and SVM references reported above. Although these offline references operate directly on the ground-truth neural activity, the results show that RNDC-Lab can incorporate real-time calcium-imaging processing and online model updating while maintaining competitive decoding performance.

#### 4.5.3 Decoding performance and verification of functional modules

We constructed a testing framework to validate the decoding performance of the system built by RNDC-Lab on various datasets and to verify the correctness of the RNDC-Lab framework with its three main functional modules. Recall that the proposed RNDC-Lab framework contains three main functional modules: (1) the RNDAE subsystem, which is NDSEP in this work; (2) the decoding model preparation subsystem, which is NeuroGRS in this work; and (3) the decoding model inference subsystem, which is NGSynth in this work. The testing framework can be used to test the current RNDC-Lab system with NDSEP, NeuroGRS, and NGSynth in this work and to test future RNDC-Lab system updates with new components, such as a new inference system or model preparation algorithm. This experiment is designed to verify the functional consistency of the integrated RNDC-Lab pipeline by comparing decoding performance using ground-truth neuron spiking signals and NDSEP-extracted neuron spiking signals with an MLP decoder and RNDC-Lab in dynamic mode. Through these comparisons, the experiment assesses whether information loss in the front-end signal extraction stage remains acceptable for downstream decoding.

Using the testing framework described above, this experiment iteratively runs RNDC-Lab with a sequence of *N* simulated calcium imaging data streams. In this experiment, we use *N* = 27. The prediction results from the classifier implemented in the *model*_*inference* actor are recorded first and used to calculate the whole-system classification metrics by comparing with the given ground-truth behavior labels for the input calcium imaging frames. The extracted neuron spiking signals from the NDSEP system are recorded in a separate file and later used to train and evaluate an MLP decoder.

Classification results of the MLP decoder on the neuron spiking signals extracted by the NDSEP subsystem can be used to assess the decoding performance of the extracted signals by comparison with the results of RNDC-Lab in dynamic mode. The quality of the NDSEP-extracted neuron spiking signals can also be assessed by comparing MLP decoding performance on the ground-truth neuron spiking signals and the corresponding NDSEP-extracted signals.

In this experiment, we use the N90 dataset described in Section 4.1.4. A total of 27 simulated calcium imaging data streams are employed with different levels of noise, motion, and rotation. Each data stream contains 1200 frames. We configure RNDC-Lab to operate in dynamic mode with an updating rate of *updateRate* = 300.

We train the MLP decoder on the first 300 samples and test it on the last 700 samples of either the ground-truth or NDSEP-extracted neuron spiking signals. The frames preceding the last 700 frames are not involved in inference since the system is in its initialization stage across those frames—i.e., collecting the first training batch and training the first compact DNN classifier.

As shown in Table 11, we report decoding results for the MLP decoder using either ground-truth or NDSEP-extracted neuron spiking signals, together with the results of RNDC-Lab in dynamic mode, for simulated frames with three different noise levels: η∈{0.1, 0.2, 0.3}, without motion or rotation. The results in Table 11 show that RNDC-Lab in dynamic mode achieves balanced accuracy similar to that of the corresponding MLP decoder.

**Table 11:** Neural decoding results of the MLP decoder using ground-truth and NDSEP-extracted neuron spiking signals, and of RNDC-Lab in dynamic mode under different noise levels.

	*MLP*_*decoder*	*dynamic*_*mode* (updatingRate = 300)
groundTruthNeuronSpikingSignal	0.995 ± 0.01	1 ± 0
NDSEP neuronSpikingSignal (η=0.1)	0.919 ± 0.006	0.925 ± 0.006
NDSEP neuronSpikingSignal (η=0.2)	0.922 ± 0.004	0.912 ± 0.004
NDSEP neuronSpikingSignal (η=0.3)	0.926 ± 0.006	0.915 ± 0.007

The MLP decoder achieves balanced accuracy near 1 using the ground-truth neuron spiking signals and approximately 0.92 using the NDSEP-extracted signals. We can draw two conclusions from these results: first, the final decoding performance remains similar across the different noise levels introduced in the simulated calcium imaging frames. Second, the extracted neuron spiking signals from NDSEP incur some degree of information loss compared to the ground-truth neuron spiking signals. It is anticipated that this information loss occurs in the neuron detection and neural signal extraction parts of the NDSEP subsystem.

#### 4.5.4 Decoding performance under motion and rotation

We report the balanced accuracy of the *dynamic*_*mode* on 27 simulated calcium imaging data streams to demonstrate how motion and rotation affect its decoding performance. This experiment evaluates the full RNDC-Lab pipeline under degraded input conditions. Specifically, we examine how motion, rotation, and noise affect the final decoding performance after front-end processing, signal extraction, and model inference are integrated into the same workflow. The results are generated using the same experimental strategy and settings described in Section 4.5.3.

Results are shown in three tables: Tables 12–14 with three different noise levels: η = 0.1, η = 0.2, and η = 0.3, respectively. Each table has three rows with increasing α values: 0, 3, 6, and three columns with increasing β values: 0, 1, 2. As described in Section 4.1, α is a parameter that controls the degree of random motion, and β is a parameter that controls the degree of random rotation.

**Table 12:** Decoding results of *dynamic*_*mode* on the N90 dataset with different degrees of motion and rotation under noise level η = 0.1.

η = 0.1	β = 0	β = 1	β = 2
α = 0	0.925 ± 0.006	0.873 ± 0.008	0.872 ± 0.009
α = 3	0.908 ± 0.008	0.911 ± 0.007	0.896 ± 0.004
α = 6	0.921 ± 0.01	0.905 ± 0.006	0.878 ± 0.006

When β increases—in Tables 12–14—there are some decreases in balanced accuracy in *dynamic*_*mode* with the same α value. This is more obviously seen in Table 14, when α = 6. This indicates that RNDC-Lab can be impacted moderately by rotation. Overall, the proposed system gives stable decoding performance on different degrees of frame rotation.

In Tables 12, 13, when α increases with the same β value for all three noise levels, there is no drop in the balanced accuracy of RNDC-Lab. This shows that RNDC-Lab performs well with all degrees of motion with a noise level equal to or under η = 0.2. In Table 14, when β∈{1, 2}, we can see that the balanced accuracy in *dynamic*_*mode* drops with increasing α. This shows that RNDC-Lab can be impacted by motion under a high noise level of η = 0.3 and a rotation level equal to or higher than β = 1.

**Table 13:** Decoding results of *dynamic*_*mode* on the N90 dataset with different degrees of motion and rotation under noise level η = 0.2.

η = 0.2	β = 0	β = 1	β = 2
α = 0	0.912 ± 0.004	0.872 ± 0.011	0.870 ± 0.007
α = 3	0.922 ± 0.004	0.889 ± 0.008	0.896 ± 0.007
α = 6	0.911 ± 0.007	0.893 ± 0.008	0.890 ± 0.006

**Table 14:** Decoding results of *dynamic*_*mode* on the N90 dataset with different degrees of motion and rotation under noise level η = 0.3.

η = 0.3	β = 0	β = 1	β = 2
α = 0	0.915 ± 0.007	0.901 ± 0.006	0.874 ± 0.005
α = 3	0.921 ± 0.006	0.794 ± 0.012	0.850 ± 0.006
α = 6	0.928 ± 0.005	0.862 ± 0.009	0.842 ± 0.008

By comparing these three tables, we can see that noise impacts the proposed system when the input frames have a high degree of motion and rotation. Noise does not impact the system under a no-motion and no-rotation situation, which can also be observed from Table 11. Overall, RNDC-Lab performs in a stable manner under different levels of noise, motion, and rotation. The 0.925 to 0.842 drop in balanced accuracy is acceptable under the highest noise level η = 0.3, the highest motion level α = 6, and the highest rotation level β = 2.

### 4.6 Decoding and runtime experiment on real-world calcium imaging data

We further evaluate RNDC-Lab using real, preprocessed calcium imaging data from the MSN dataset (Section 4.1.5). The purpose of this experiment is to evaluate real-time decoding and model updating under concept drift using real *in vivo* data, rather than the complete raw-data workflow. Preprocessed neural traces are used to minimize the confounding effects of motion and thereby focus the evaluation on changes in the neural representation and the corresponding model-updating behavior.

The neural traces and behavioral labels are directly sent into the preprocessed-data pipeline. Static and dynamic modes use the first 500 frames for initial training, while the dynamic mode updates the model every additional 500 frames. Prediction is evaluated over frames 501–9,000.

As shown in Figure 9, the selected neurons exhibit clear temporal variations in activity throughout the recording. Because the behavioral labels and decoding outputs are binary, they are visualized as discrete class-0/class-1 tracks rather than continuous trajectories, making the prediction patterns less straightforward to assess visually. Nevertheless, the quantitative results show that the dynamic mode achieves a higher overall prediction accuracy of 0.649 than the static mode, which achieves 0.608, indicating improved decoding performance with model updating for this real-data experiment.

**Figure 9:**
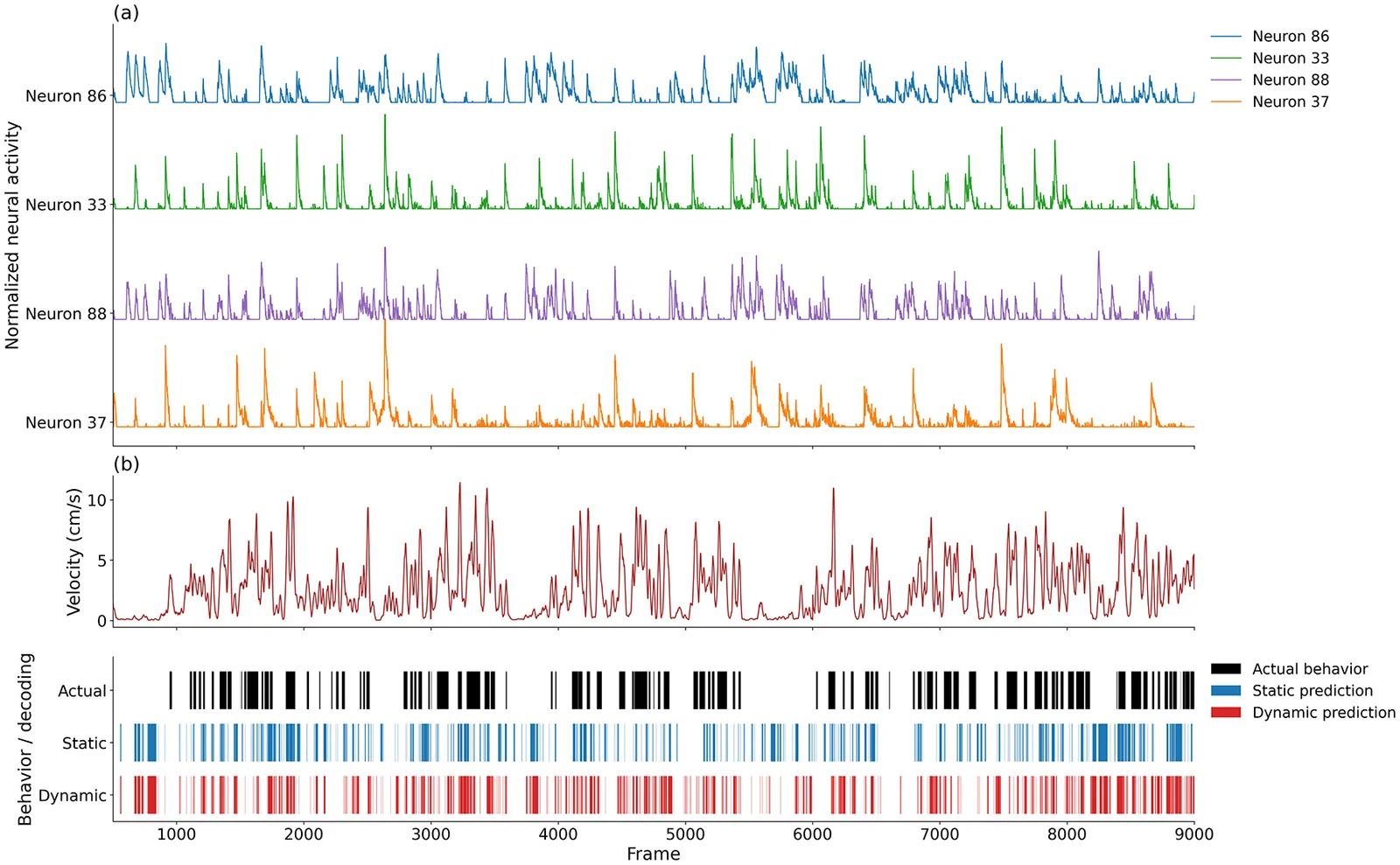
Representative MSN neural activity, locomotor velocity (cm/s), actual behavioral labels, and RNDC-Lab static and dynamic decoding predictions over frames 501–9,000. Four active neurons with the highest temporal variance are shown after standardization and vertical offset. Colored segments indicate class 1 and blank segments indicate class 0. **(a)** Representative MSN neural activity. **(b)** Actual locomotor velocity and RNDC-Lab decoding.

Runtime performance is evaluated at the actor level for the inference and training graphs over 10 independent runs under both static and dynamic modes. For each run, the maximum, mean, and minimum runtimes are calculated over all invocations of each actor, and the reported values are the averages of the corresponding statistics across the 10 runs.

As shown in Table 15, the inference graph maintains low average runtime under both modes. The mean runtime summed across all actors is approximately 11.07 ms in static mode and 11.17 ms in dynamic mode, indicating that dynamic model updating introduces little additional overhead into the inference path. The approximately 10 ms mean runtime of the preprocessed_reader is primarily determined by the configured input frame rate rather than by data-loading computation. This actor regulates the arrival rate of neural samples to emulate streaming input and prevent the entire dataset from being processed immediately, allowing the concurrent model-updating behavior to be evaluated.

**Table 15:** Runtime of actors in the inference graph under static and dynamic modes.

Actor name	Static runtime (ms)			Dynamic runtime (ms)		
	Max	Mean	Min	Max	Mean	Min
preprocessed_reader	2,356.986	10.404	0.001	10.373	10.125	0.003
signal_accumulator	0.158	0.002	0.000	3750.531	0.425	0.000
snd_dsg_mpi	0.016	0.000	0.000	0.026	0.001	0.000
rec_dsg_mpi	0.027	0.001	0.001	0.045	0.002	0.001
MI_NGSynth	5,390.532	0.664	0.007	4,799.655	0.617	0.007
sum of all actors	7,747.721	11.072	0.009	8,560.630	11.170	0.011

Table 16 shows that model synthesis dominates the execution time of the training graph. In static mode, MP_NeuroGRS + synthesis is executed only once per run for the initial model. Therefore, the maximum, mean, and minimum synthesis runtimes are identical within each run, resulting in the same reported average value of approximately 9.90 s across the 10 runs. In dynamic mode, additional models are generated as new samples become available. Since successive updates use increasing amounts of accumulated training data, the synthesis runtime varies across updates, with maximum, mean, and minimum runtimes of approximately 10.55 s, 5.88 s, and 3.83 s, respectively.

**Table 16:** Runtime of actors in the training graph under static and dynamic modes.

Actor name	Static runtime (ms)			Dynamic runtime (ms)		
	Max	Mean	Min	Max	Mean	Min
MP_NeuroGRS+synthesis	9,898.032	9,898.032	9,898.032	10,549.894	5,883.446	3,828.162
rec_dsg_mpi	7.569	7.569	7.569	8.032	7.515	7.240
snd_dsg_mpi	0.005	0.005	0.005	0.009	0.006	0.004
active	0.374	0.003	0.003	0.382	0.003	0.003
sum of all actors	9,905.979	9,905.609	9,905.609	10,558.317	5,890.970	3,835.409

As described previously, model synthesis is performed concurrently with inference, so RNDC-Lab continues decoding with the most recently available model while an updated model is being prepared, avoiding direct inference delay.

## 5 Conclusion

This paper has introduced RNDC-Lab, a software framework designed for real-time neural decoding systems using calcium imaging data. Leveraging this framework, we have developed a novel real-time neural decoding system that integrates three complementary tools, NDSEP, NGSynth, and NeuroGRS, which have been developed in prior research to address important sub-problems related to neural decoding. NDSEP serves as the front-end RNDAE (Real-time Neuron Detection and Activity Extraction) subsystem for calcium imaging preprocessing tasks, including neuron detection, motion correction, and neural signal extraction. NeuroGRS functions as the model preparation subsystem, generating efficient compact DNN models optimized for real-time inference. NGSynth operates as the back-end model inference subsystem. RNDC-Lab provides a systematic framework for interfacing, integrating, and optimizing important sub-tasks, including neuron detection, motion correction, signal extraction, neural decoding model pre-training, dynamic model training, and neural decoding inference. While the components within NDSEP, NGSynth, and NeuroGRS are used to concretely demonstrate the capabilities of RNDC-Lab in this paper, the rigorous dataflow-based architecture of RNDC-Lab enables these components to be selectively replaced with alternative modules that provide the same general functionality using different algorithms or implementations (e.g., a new module for motion correction or model retraining). This makes RNDC-Lab a powerful tool for experimenting with novel aspects of neural decoding in the context of an overall real-time framework.

The experimental capabilities of RNDC-Lab are further enhanced by four new datasets—the Brain Phantom, N80, N90 and Reward Learning (for multiclass classification) datasets—that we introduce in this work as companion resources for working with RNDC-Lab.

RNDC-Lab employs innovative methods, such as dynamic loading and parallel dataflow modeling, to efficiently integrate the component subsystems while supporting dynamic model updating functionality. Extensive experiments demonstrate the model-updating capability of RNDC-Lab and characterize its associated trade-offs involving decoding performance and execution time. Moreover, we show that the demonstrated neural decoding system can successfully extract meaningful information from both simulated and real-world datasets.

The static and dynamic operating modes of RNDC-Lab provide different trade-offs between real-time performance and adaptation to concept drift. In static mode, the demonstrated system exhibits a deadline miss rate of only 0.67% for the 100-ms frame-processing constraint. Dynamic mode enables model updating in response to concept drift, but this additional capability incurs substantial computational overhead, resulting in a deadline miss rate of 19.19% in the current implementation. Thus, dynamic mode is most relevant in operating environments where significant changes in the underlying neural data distribution make adaptation important enough to justify the additional computational cost. The present results should therefore not be interpreted as demonstrating that dynamic mode is generally preferable to static operation; rather, they demonstrate the feasibility of incorporating model adaptation within a real-time neural decoding system and expose the associated performance trade-offs. An important capability of RNDC-Lab is to enable such trade-offs to be identified and quantitatively evaluated, thereby providing information that can guide subsequent optimization of the algorithms and their implementations toward more comprehensive satisfaction of real-time constraints.

Important directions for future work include improving both the efficiency and selectivity of dynamic model updating, and evaluating the complete RNDC-Lab workflow using raw *in vivo* calcium imaging recordings. In particular, robust methods are needed to detect concept drift and determine when model updating is likely to provide sufficient benefit to justify its computational overhead. Such methods could enable the system to remain in the lower-overhead static mode when the current model remains effective and invoke dynamic updating selectively when changes in the underlying data warrant adaptation. RNDC-Lab can also help direct optimization effort by enabling integrated exploration of algorithms, module (dataflow actor) implementations, and system-level design methods to identify and address the primary sources of deadline violations. Further work is needed both to reduce the latency associated with model updating and to evaluate more comprehensively the conditions under which dynamic adaptation provides measurable advantages in decoding performance.

A limitation of the present study is that RNDC-Lab is evaluated as a real-time neural decoding framework without integration with physical stimulation or feedback hardware. Integration with such hardware to form a complete closed-loop system remains a useful direction for future work. The current experiments focus on real-time performance on a commodity computing platform rather than embedded deployment. Extending RNDC-Lab to more resource-constrained platforms would require further evaluation and optimization of runtime, memory usage, energy consumption, and platform-specific implementation, particularly for applications involving implanted or otherwise resource-limited neural decoding systems.

The proposed system integrates RNDC-Lab with NDSEP, NGSynth, and NeuroGRS. In this work, our experiments center on one-photon calcium imaging, which is widely used to study neural activity in behaving animals. Both the brain phantom and MSN datasets were acquired using one-photon excitation. However, it is expected that the proposed system can also be used for two-photon excitation with customization for specific devices. This can be achieved by providing new dataflow actors. In fact, the original study of NDSEP has already been validated on two-photon calcium imaging data. Our future work will include further validation on two-photon calcium imaging.

## Data Availability

The raw data supporting the conclusions of this article will be made available by the authors, without undue reservation.
